# Fe‐2,5‐furandicarboxylate Metal Organic Frameworks with Rare Building Blocks and a Ligand that can be Biomass‐Derived as Effective Catalysts for Selective Nitroarene Reduction

**DOI:** 10.1002/cssc.202500120

**Published:** 2025-04-02

**Authors:** Satarupa Das, Omar Al‐Miqdadi, Wai Wing Cheng, Jeremiah P. Tidey, Marc Walker, Gaurav C. Pandey, Richard I. Walton

**Affiliations:** ^1^ Department of Chemistry University of Warwick Coventry CV4 7AL UK; ^2^ Department of Physics University of Warwick Coventry CV4 7AL UK; ^3^ Warwick Manufacturing Group University of Warwick Coventry CV4 7AL UK

**Keywords:** Metal-organic framework, iron, 2,5-furan dicarboxylic acid, rod-like SBU, heterogeneous catalysis

## Abstract

We report two three‐dimensional metal‐organic frameworks constructed from Fe^3+^ and the ligand, 2,5‐furandicarboxylate (FDC) that can be derived from biomass. One contains an unprecedented infinite‐rod‐shaped building unit, and the other is the first crystalline framework of FDC that contains solely iron in the metal nodes. The materials are formed as microcrystals and their structures are determined using 3D‐electron diffraction with the bulk confirmed by powder XRD. UOW‐7, NaFe_5_O_3_(FDC)_4_(CH_3_COO)_2_(H_2_O) is a bimetallic structure with acetate as co‐ligand, constructed from infinite chains of iron octahedra, wherein tetramers comprising edge‐sharing pairs linked by corner sharing octahedra are crosslinked by FDC ligands. In contrast, UOW‐8, [Fe_2_O(FDC)_2_(H_2_O)_2_]⋅(H_2_O)_4_ contains a rare form of tetrameric building unit, cross‐linked by FDC, and having Fe‐bound water as well as occluded water. The materials crystallise under hydrothermal conditions and are water‐stable coordination polymers with no measurable free pore space. The catalytic ability of UOW‐7 and UOW‐8 is, nevertheless, established in the reduction of 4‐nitrophenol to 4‐aminophenol by borohydride, where both act as recyclable, catalysts to give ~100 % yield of the product without use of precious metals. UOW‐8 is found to have the more favourable reaction kinetics, likely due to the presence of surface Lewis acidic Fe^3+^ centres that enhance substrate binding.

## Graphical abstract

Two metal‐organic frameworks, UOW‐7 and UOW‐8, crystallise from iron and the ligand 2,5‐furandicarboxylate under aqueous conditions. The ligand precursor can be sourced from biomass, providing an attractive alternative to common fossil‐derived ligands, while the new MOFs show high activity and recyclability for the reduction of nitrophenol (NP) to aminophenol (AP) in water.

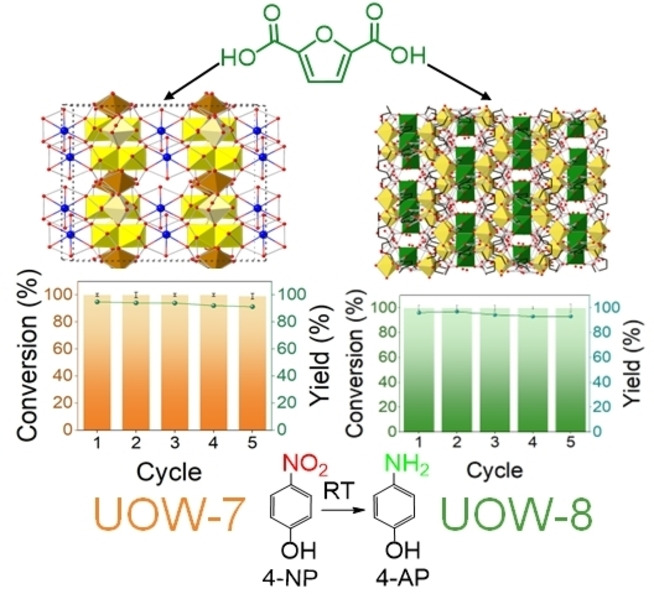

## Introduction

Metal−organic frameworks (MOFs) have emerged as an intensely studied research field over the past two decades.^[1]^ In contrast to conventional inorganic porous solids like zeolites that exhibit comparatively limited structural diversity, MOFs stand out for their vast array of potential structures from using a wide variety of metallic elements from across the Periodic Table combined with ligands with a wide range of geometries and connectivities. MOFs also offer the possibility of chemical functionalisation, making use of the organic chemistry of the ligands or by post‐synthesis exchange of either ligands or metal cations. While MOFs offer tunability and ease of functionality, their long‐term stability is often limited, especially towards water, has impeded their extensive use in, for example, environmental pollutant capture, industrial gas separations and catalysis.[^2^, ^3^, ^4^] Only recently have systematic features of MOF structural chemistry that lead to water stability been identified, including the use of high‐valent metal cations partially condensed in clusters or rods, highly connected by rigid aromatic linkers.[^5^, ^6^, ^7^]

In the field of MOFs, significant research has been focussed on Fe‐containing materials, in large part owing to the non‐toxic nature and ready availability of iron. In terms of practical applications, the Lewis acidity of Fe^3+^ gives rise to strong ligand binding, imparting stability to many Fe‐MOFs in both organic solvents and aqueous solutions, while the redox chemistry of iron is relevant to heterogeneous catalysis. There have been a significant number of iron MOFs already reported.[^8^, ^9^, ^10^] The trimeric [Fe_3_(μ_3_‐O)(−COO)_6_] and infinite chain [Fe(OH)(−COO)_2_]_n_ are the most prevalent building blocks seen in the iron containing MOFs, with the former seen in materials such as in the MIL‐88 series, MIL‐100 and MIL‐101, and the latter in MIL‐53 and MIL‐68.

In recent years, there has been a growing effort to explore and harness biomass as an economical, renewable, and readily available feedstock or precursor for various chemical processes and applications.[^11^, ^12^] This idea should naturally be extended to the synthesis of ligands for MOFs, if the materials are to be used in future applications, where sustainably sourced precursors will be increasingly important. A variety of valuable chemicals can be obtained from lignocellulosic biomass, with the dicarboxylic acid, 2,5‐furandicarboxylic acid (H_2_FDC), being a significant product that is obtainable through the selective oxidation of biomass‐derived 5‐hydroxymethylfurfural (5‐HMF).[^13^, ^14^] The use of its deprotonated form, 2,5‐furandicarboxylate (FDC), as a ligand for metal‐organic frameworks (MOFs) is an attractive concept, especially bearing in mind that benzene dicarboxylates, such as terephthalate (benzene‐1,4‐dicarboxylate) and isophthalate (benzene‐1,3‐dicarboxylate), which are currently ubiquitous in MOF chemistry, are derived from fossil resources via oxidation of xylenes.[^15^, ^16^] Despite future environmental advantages, only a comparatively small number of FDC‐based MOFs have been successfully synthesised and characterised to date, with examples including those of aluminium,^[17]^ zirconium,^[18]^ and of rare‐earths.^[19]^ It is important to note that the industrial‐scale production of FDCA from biomass is soon to be realised,^[20]^ which will allow a more plentiful supply of this ligand precursor for MOFs.

Herein, we report the synthesis and characterisation of two iron MOFs with 2,5‐furan dicarboxylate acid as ligand. These represent the first crystalline MOFs containing FDC and solely iron as the high valent transition‐metal cation. To our knowledge the only relevant examples in the literature are an amorphous iron‐organic gel,^[21]^ and a mixed‐metal MOF containing the trimeric Fe_2_Co secondary building unit (SBU) prepared from pre‐formed clusters in the Fe_2_Co(μ_3_‐O)(CH_3_COO)_6_ precursor.[^21^, ^22^] The new materials show hydrothermal stability and we have assessed their catalytic ability in the reduction of 4‐nitrophenol (4‐NP) to 4‐aminophenol (4‐AP) as a model reaction. Para‐nitrophenol (4‐NP) poses a significant threat to both human health and the environment, being a hazardous and toxic organic contaminant, while the resultant product 4‐aminophenol (4‐AP) serves as a useful intermediate in the production of dyes and pharmaceuticals.^[23]^ Because of its extreme hazards towards human health, 4‐NP is listed on the U.S. Environmental Protection Agency′s list of priority pollutants for environmental remediation,^[24]^ and innovative ways for degradation of nitrophenols are the focus of much attention.^[25]^ Our findings underscore the significance of a strategic design for functional Fe‐based MOFs making use of biomass‐derived ligands.

## Results and Discussion

Two novel iron‐based MOFs, namely UOW‐7 and UOW‐8, were successfully synthesised using either hydrothermal or reflux conditions, respectively. Both MOFs are formed as polycrystalline powders containing crystallites too small for conventional crystallographic analysis and the structures were instead solved by 3D electron diffraction (3DED; Table S1‐S2). Precise experimental details are provided in the Supplemental Information, Experimental Section.

**Figure 1:**
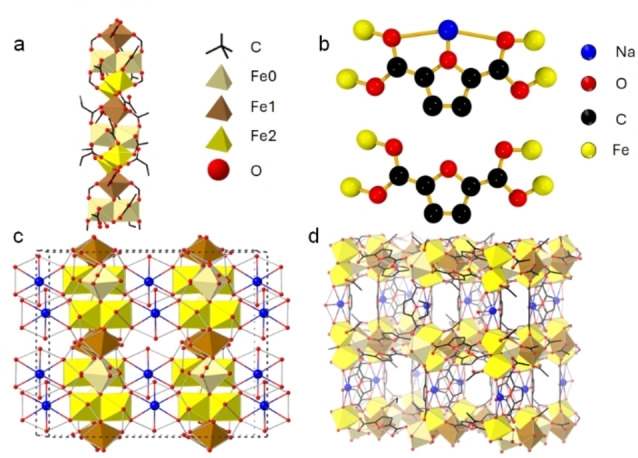
(a) Representation of the infinite iron chain in UOW‐7, (b) binding modes of FDCA in UOW‐7, (c)The iron SBU connectivity with the sodium cation (d) 3D representation of UOW‐7

UOW‐7 was synthesised from a hydrothermal reaction employing iron acetate and 2,5‐furan dicarboxylic acid in the presence of a water/acetic acid solvent mixture. The material crystallises in the orthorhombic space group, *Cmce*, with the chemical formula Fe_5_NaO_3_(FDC)_4_(CH_3_COO)_2_(H_2_O) and hence contains only Fe^3+^. The core backbone of UOW‐7 is a unique iron‐oxide chain (Figure 1a). This contains a tetramer of pairs of edge‐shared octahedra sharing a single unique oxide that are linked by *trans* corner‐sharing octahedra to give an infinite rod SBU. The chain is comprised of three crystallographically distinct iron atoms connected by bridging oxide anions. Each iron centre within the chain is octahedrally coordinated: Fe0 coordinates with one fully deprotonated acetate ion, for which the other oxygen coordinates Fe2, three distinct FDC^2−^ ions (the binding modes of FDC are displayed in Figure 1b), and links to Fe1and Fe2 by μ_3_‐O and μ_4_‐O, respectively, while Fe1 coordinates with four equatorially arranged FDC anions and connects to neighbouring iron atoms via distinct μ_3_‐O atom. Similarly, Fe2 is also coordinated to three unique FDC ions, one acetate ion, and the remaining coordination is completed by bridging μ_3_‐O and μ_4_‐O.

**Figure 2:**
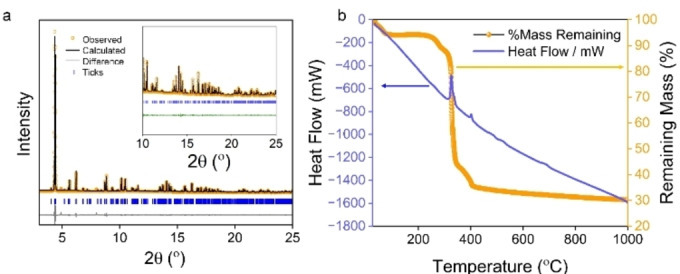
Characterisation of UOW‐7. (a) Pawley fit of the PXRD of UOW‐7. Inset shows an expanded view of the fitting (see Table S3 for refined lattice parameters). (b) TGA of as‐synthesised UOW‐7

Overall, the chains can be described as tetrameric clusters of FeO_6_ octahedra, wherein two edge sharing dimers are rotated through 90 degrees about a common, central oxide ion in a tetrahedral geometry. The tetramers are then linked together by singular FeO_6_ octahedra in a *trans*, corner‐sharing arrangement about further oxide ions, with each terminus in a triangular planar arrangement with two Fe centres of a given dimer. To our knowledge, this chain type has not been reported in any other MOF.^[26]^ The structural integrity of UOW‐7 is reinforced by sodium atoms, which are connected to adjacent Fe0 atoms by two μ_2_‐O atoms derived from distinct FDC ions. (Figure 1c) Pairs of FDC ligands arrange in a buckled planar manner wherein furanyl oxygens direct toward one another to form conjugated crown‐ether‐like pockets in which additional Na_+_ ions are bound. The iron chains are connected to each other by FDC ions, to give a three‐dimensional architecture (Figure 1d). The bulk structure is confirmed by a Pawley fit of the powder XRD, based upon the output from 3DED, which shows excellent agreement with the simulated powder pattern of the solved structure (Figure 2a). The structural model for UOW‐7 is corroborated by EXAFS (Figure S1‐S5, Table S5‐S7, IR spectroscopy (Figure S7‐S8, Table S9, see SI for detail analysis) while the Fe K‐edge XANES spectrum (Figure S6) confirms the expected Fe^3+^, also consistent with bond valence sums, X‐ray fluorescence spectra confirm the presence of Na, while ICP‐OES confirms the bulk Fe : Na ratio (measured 5.4 : 1, expected 5 : 1).

The TGA (Figure 2b) shows thermal stability to around 300 °C, which is preceded by a loss of surface water that commences immediately on heating (7.3 %). The complete combustion that follows to give Fe_2_O_3_ and Na_2_O is accounted for by ligand (FDC and acetate) and crystal water loss (expected 63.8%, observed 63.0%). Thermodiffraction (Figure S9) shows that crystallinity is maintained before collapse with little change in structure, after which an amorphous phase is produced. The porosity of the structure is, however, negligible. Void space was calculated by generating an electron density isosurface using CrystalExplorer(^[27]^ at an isovalue of 0.0003 au (established to be the most suitable for mapping available space within molecular crystals). As shown in Figure S10, the channels running parallel to *b*, are constricted by extremely narrow openings with a diameter of ~0.5 Å, too small for any atom or molecule to pass through. The lack of any measurable porosity by nitrogen adsorption is in agreement with this. (Figure S11)

**Figure 3:**
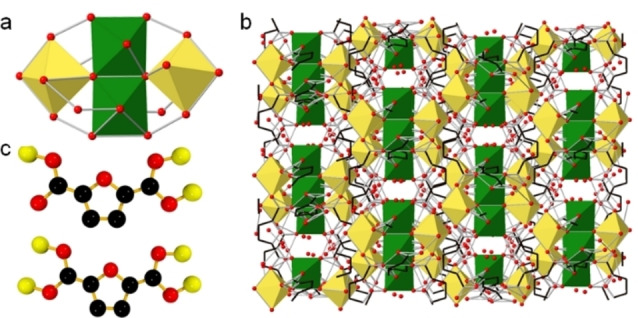
(a) Schematic representation the Fe_4_O_2_ tetrameric building unit connected by FDC units in UOW‐8, (b) binding modes of FDC ligand in UOW‐8 (c)3D representation of UOW‐8

UOW‐8 is synthesised by a reflux reaction in water using iron chloride hexahydrate as a precursor in the presence of sodium hydroxide. Structure solution reveals the chemical formula to be [Fe_3_(FDC)_2_O(H_2_O)_2_]⋅(H_2_O)_4._ The material crystallises in monoclinic space group, *C*2/*c*. The SBU here contains the unusual tetrameric Fe cluster [Fe_4_(μ_3_‐O)_2_(COO)_8_] (Figure 3a). The core [Fe_4_(μ_3_‐O)_2_]^8+^ comprises four Fe(III) ions arranged in a “butterfly” configuration. This core incorporates two μ_3_‐O ions, each linking three Fe(III) ions and forming the “wings” of the “butterfly”. The Fe2 and Fe3 atoms occupy the central positions, while Fe1 and Fe4 occupy the ”wingtip“ positions. Figure 3b shows the 3D framework. IR spectroscopy (Figure S7‐S8, Table S9 and EXAFS ((Figure S1‐S5, Table S7, support the structural model for UOW‐8 and the Fe K‐edge XANES spectrum ( Figure S6) confirms the expected Fe^3+^, also deduced from bond valence sums.

**Figure 4:**
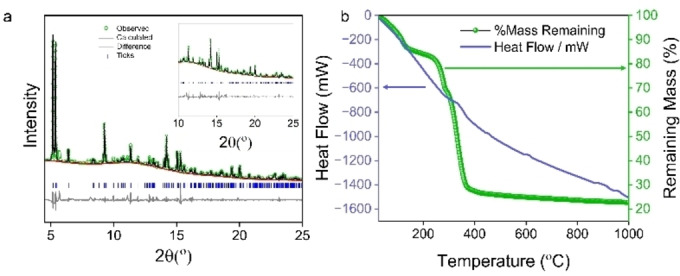
Characterisation of UOW‐8. (a) Pawley fitted XRD of UOW‐8. Inset shows the fitting at higher two theta angle (see Table S2 for refined lattice parameters). (b) TGA of as‐synthesised UOW‐8

The same tetrameric Fe cluster has only recently been reported in a MOF constructed from terephthalate and diaminotriazole ligands^[28]^ and is only catalogued in a few other MOF materials for other metals.^[29]^ Each metal centre forms coordination bonds with the oxygen atoms of four distinct carboxylate groups from four distinct linker molecules. This interaction results in the completion of an octahedral coordination geometry around the metal centres. The ligands are bound to the metal in a bidentate μ_2_‐η^1^: η^1^ fashion (Figure 3c). Two of the iron octahedra that are placed horizontally contain terminally bound waters. One metal oxygen cluster connects to the other via μ_3_‐η^2^: η^1^fashion. The [Fe_4_(μ_3_‐O)_2_]^8+^ core structural unit can adopt chair‐ and boat‐like conformations.^[30]^ In the chair form, strain is minimised and so predominates in the MOFs that contain the same tetranuclear core and is also evident here for UOW‐8 (Figure S12). As observed in Figure 4a, the Pawley fit of the powder XRD of UOW‐8 shows the structure to be in good agreement with the measured pattern, confirming the bulk identity of the sample.

TGA of UOW‐8 (Figure 4b) shows it to have lower thermal stability than UOW‐7, and a total observed mass loss of 77.40 % compared to the expected value around 70.63 %. We attribute the discrepancy of mass loss in the TGA to a large amount of surface water associated with the material, evidenced by mass loss that commences immediately upon heating. Thermodiffraction (Figure S13) shows shifts in Bragg peaks followed by a structural transition with loss of the crystal water, before collapse occurs to give an amorphous material, which corresponds to the combustion of the organic ligand. The potential porosity was calculated in the same manner as for UOW‐7 (Figure S14), except for here removing non‐Fe‐bound water from the structure to simulate an activated or dynamic system. The removal of pore water leaves behind modest slabs of void, capable of accommodating a few gas molecules, yet these are connected by only very narrow channels that again seem too small to permit the passage of even very small molecules. This is further evidenced by a failure to obtain a surface area from experimental nitrogen adsorption measurements (Figure S15). We note that no void space could be found when employing an iso value of 0.0003 au without omission of the pore water.

Scanning electron microscopy shows that particles of UOW‐7 have a prominent, uniform, cuboid structure, whereas anisotropic rod‐like particle morphology is seen for UOW‐8 (Figure S16). In order to assess stability of the materials in aqueous solvents, samples were soaked in water at room temperature where even after a week they showed no loss in crystallinity by powder XRD (Figure S17).

The redox catalytic activity of UOW‐7 and UOW‐8 was quantitatively assessed in the reduction of 4‐nitrophenol (4‐NP) to 4‐aminophenol (4‐AP) using excess sodium borohydride (NaBH_4_) as the reducing agent under basic conditions. The reaction kinetics was monitored using UV‐vis absorption spectroscopy where the colour change of the 4‐NP solution from light yellow to bright yellow was characterised by a shift in the absorption maximum from 317 nm to 400 nm, indicating the formation of nitrophenolate (Figure S18). After this, the solution gradually turns colourless and a new absorption emerges at around 295 nm, corresponding to 4‐AP. The evolution of the absorption spectra at 400 nm was tracked to observe the reaction progress. The concentration of *p*‐nitrophenol at time (*t*) is denoted as *C*_t_, and the initial concentration of nitrophenols at *t*=0 is regarded as *C*_0_. The *C*_t_/*C*_0_ is measured from the relative intensity of the absorbance (*A*_t_/*A*_0_). The linear relationship of ln(*C*_t_/*C*_0_) versus *t* indicates that the reduction of 4‐NP by these MOFs follows pseudo first‐order kinetics. In the absence of a catalyst, the absorption peak at 400 nm remains constant for a prolonged time, indicating NaBH_4_ alone is incapable of any significant reduction of 4‐NP (Figure S18). Similarly, the MOF catalysts without NaBH_4_ also show negligible activity for the reduction of 4‐NP.

**Figure 5:**
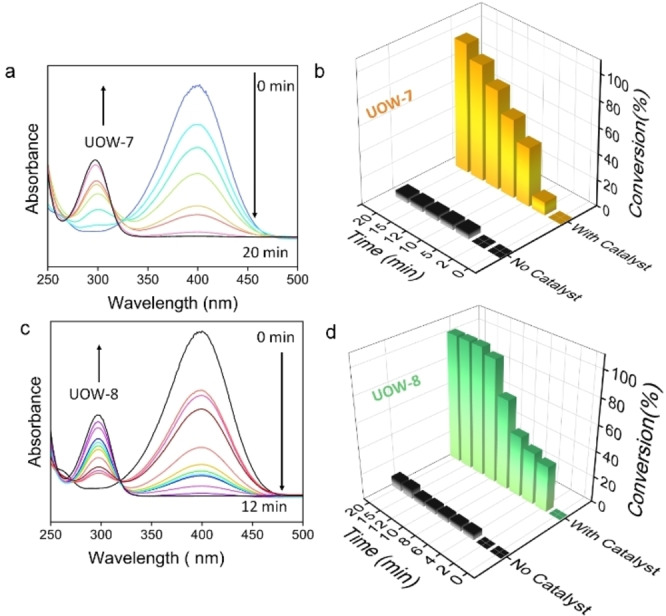
Time‐dependent UV‐vis spectral changes in p‐nitrophenol and corresponding catalytic efficiency determination for reaction with NaBH_4_ in the presence and absence of MOF catalyst UOW‐7 (a, b) and UOW‐8 (c, d)

Figure 5(a) &(b) show the time dependent UV spectra for the reaction of 4‐NP with NaBH_4_ using UOW‐7 as the catalyst, with 100 % conversion being observed after 20 mins of reaction. When UOW‐8 was employed, the catalyst completely reduced 4‐NP in 12 minutes (Figure 5(c) &(d). The identification of isosbestic points indicates that 4‐NP undergoes complete conversion to aminophenol without any occurrence of side reactions. Therefore, the materials show selectivity towards the target 4‐AP product. Our observations show that UOW‐8 catalyst exhibits superior activity over UOW‐7, with less time required for the full conversion of 4‐NP. This might be ascribed to the presence of Lewis acid sites in UOW‐8, bearing in mind the bound water in the structure. The importance of Lewis acidity in enhancing the catalytic efficiency for this reaction has also been reported by Garcia and coworkers.^[31]^ For comparison we also studied the iron (III) MOF MIL‐53 that contains fully saturated octahedral Fe centres: this gave slower conversion than UOW‐8, consistent with the lack of Lewis acid sites (Figure S19). In contrast the Lewis‐acidic iron (III) MOF MIL‐100 gave similar conversion rate to UOW‐8 (Table S17, Figure S20, S23), confirming the need for Lewis acidity to effect the catalysis, although the absolute values of rate constants are lower for MIL‐100 despite its inherent internal surface area. This highlights the superior activity of UOW‐8. The rod‐like morphology of UOW‐8 compared to UOW‐7 might also be responsible for better surface to volume ratio leading to more efficient catalysis, and it is clear that each material presents a rather different crystal habit (Figure S16). Additionally, we also performed a control catalysis experiment using physical mixture of Fe_2_O_3_ and FDCA ligand under the same conditions. This gives notably poorer performance, confirming that the unique surface structure of the MOFs must be responsible for their catalytic efficiency (Figure S21).

### Kinetic Analysis

To analyse the kinetic data, the apparent rate constant (*k*_app_) was determined at a fixed concentration of 4‐NP at room temperature, while varying the NaBH_4_ concentration. In a subsequent set of kinetic measurements, the NaBH_4_ concentration was held constant, and the 4‐NP concentration was altered. Finally, the impact of reaction temperature was also explored (Table S9‐10).

**Figure 6:**
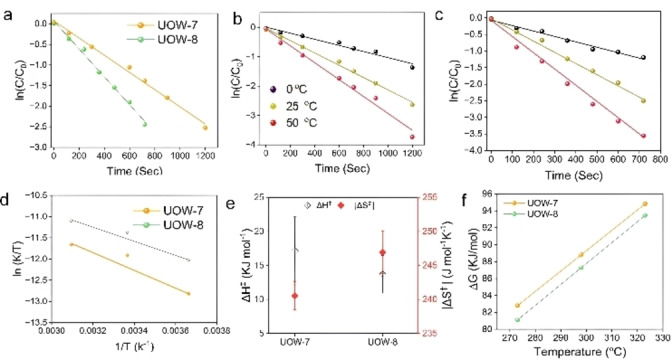
Semilogarithmic plots of: (a) 4‐nitrophenol concentration *vs* time *C* indicates reactant concentration, and *C*_0_‐ indicates the initial concentration); the reaction kinetics for (b) UOW‐7 and (c) UOW‐8 at 0, 25, and 50 °C. (d) Eyring plots for the different catalysts.(e) Δ*H* ^≠^,Δ*S* ^≠^, and (f) Δ*G* ^≠^ determined using the Eyring plots.

Figure 6a shows a semilogarithmic plot of the 4‐nitrophenol concentration versus time with each MOF catalyst at room temperature. The kinetics of 4‐NP reduction were further investigated for the two MOFs at 0 and 60 °C, additionally presented in Figures 6b and 6c. UOW‐8 exhibited consistently higher rate constants of 1.63×10^−3^, 3.39×10^−3^, and 4.86 ×10^−3^ s^−1^ compared to UOW‐7, of 0.73×10^−3^, 2.09×10^−3^, and 2.77×10^−3^ at 0, 25, and 60 °C respectively, indicating superior catalytic efficiency of the former material. (Figure 6b and c). Moreover, both of these catalysts showcase better kinetics than the physical mix of Fe_2_O_3_ and FDCA ( Figure S22, Table 12)

At high concentrations of 4‐NP, the catalyst surface is expected to experience almost complete coverage by the substrate, leading to a significant decrease in the reaction rate which means an absence of [BH_4_]^−^ ions at the active metal sites, as illustrated in Figure S24. Furthermore, the relationship between *k*_app_ and [BH_4_]^−^ concentration is nonlinear, with saturation observed at high [BH_4_]^−^ concentrations (Figure S25). This saturation suggests a competition between both reactants for the active iron sites at the catalytic surface, aligning with the operation of the Langmuir‐Hinshelwood model for catalytic 4‐NP reduction using these MOFs as catalysts.^[29]^

To further probe the reaction thermodynamics, Eyring plots were employed (Figure 6d and Tables S9‐S10) to determine the activation enthalpy (Δ*H* ^≠^) and entropy (Δ*S* ^≠^) for the conversion of reactants to the transition state species (*i. e*., the evolution of 4‐nitrophenolate) using the experimentally determined rate constants. The Δ*H* ^≠^ values for the reactions were higher in case of UOW‐8, consistent with experimental observations. The lower Δ*H* ^≠^ value for UOW‐8 (13.71 kJ mol^−1^) suggests the smallest kinetic barrier, rendering the reaction most feasible. Furthermore, the higher Δ*S* ^≠^ for UOW‐8 may contribute to increased rotational/conformational degrees of freedom associated with nitrophenol coordination (Figure 6e, Table S12).

The calculated Gibbs free energy of activation (Δ*G* ^≠^) for each MOF‐catalysed reaction was determined (Table S13) and Figure 6f clearly shows UOW‐8 to exhibit lower Δ*G* ^≠^ compared to UOW‐7. The structure of UOW‐8 has four water molecules per formula unit bound to the iron centre which, in aqueous suspension and particularly with heating, may become labile to provide unsaturated metal centres. In this way, the oxygen of the nitro group can readily coordinate to the metal centre, so becoming activated towards reduction. In addition, these Lewis acidic sites may stabilise further reaction intermediates formed during the reduction process, further lowering the energy barrier to conversion and enhancing the overall efficiency of the reaction.

The activity of UOW‐8 can be compared to the previously reported iron catalysts for the same reaction. As reported in Table S14, the MOF shows comparable performance to, and in some cases even exceed, the efficiency of other iron catalysts. Existing high‐performance materials for this purpose make use of precious metals such as Pd or Au, loaded onto Fe_2_O_3_.[^32^, ^33^] One clear advantage of UOW‐8 compared to these is that no precious metals are required and no further modifications are needed to obtain a viable catalyst.

**Figure 7:**
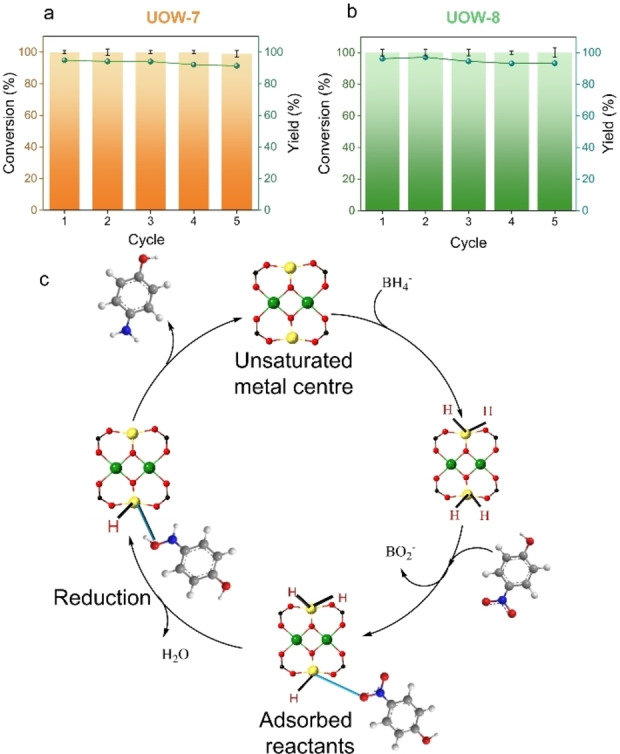
(a) and (b) plots of the recycling test activity for UOW‐7 and UOW‐8, respectively. (c) Proposed mechanism for the nitrophenol reduction at the MOF catalyst.

Assessing the stability and recyclability of the catalyst is crucial for their practical use. The recyclability of the MOFs was monitored over five consecutive reactions. Following each reaction, the catalyst was recovered and dried and subsequently reused without any additional treatment. As observed from figure 7a and b, both materials retain their catalytic ability with negligible loss in the product yield even after 5^th^ cycle. However, UOW‐8 demonstrated better efficiency in terms of activity even after 5th cycle.

PXRD of the MOF catalysts after the cycling experiments show preservation of the structures in both cases (Figure S26). Moreover, ICP analysis of the filtered reaction solution post recyclability measurement reveals low leaching of Fe in both cases (3.4 ppm for UOW‐7 and 4.5 ppm for UOW‐8). XPS measurements were carried out to gain more insight into the changes in the catalysts brought by use. UOW‐7 was found to undergo greater surface reduction than UOW‐8, as shown in Table S15‐16, with post‐catalysis UOW‐7 having a mixture of valence states for Fe while in UOW‐8 the oxidation states are relatively unchanged (Figure S27). This propensity towards reduction of UOW‐7 might explain its lower catalytic efficiency in the model reaction, since the redox catalysis will require a facile switching between Fe^2+^ and Fe^3+^. Since the two materials have distinct crystal structures, with different topologies and ligand connectivity, their ability to accommodate changes in Fe oxidation state at the surface is likely to differ, which may also contribute to the contrasting catalytic performance. We also examined the kinetics of the second cycle which showed that UOW‐8 maintains catalytic activity better than UOW‐7 (Figure S28 and Table S17), which is consistent with the surface reduction of the latter leading to some deactivation.

A proposed mechanism for the reduction is shown in Figure 7c based on our observations and with reference to a study of the Cu‐MOF HKUST‐1 for the same catalytic process in which coordination of the nitro‐ group of 4‐NP to Lewis acid sites was proposed.^[31]^ Given that our materials do not show any porosity, and the substrate molecule is large, the catalysis must take place on the surface of the MOF crystallites. Borohydride ions engage with the MOF surface, facilitating the transfer of a hydrogen atom to the particles′ surface and 4‐nitrophenol molecules then undergo adsorption onto the MOF surface with elimination of [BO_2_]^−^. Importantly, the diffusion of reactants to/from the MOF particles, along with all adsorption and desorption equilibriums, is assumed to occur rapidly and we take the reduction of 4‐nitrophenol to be the rate‐determining step in this reaction. This reduction occurs through the interaction of adsorbed 4‐nitrophenol with surface‐hydrogen species. The subsequent detachment of the product 4‐aminophenol leads to the generation of a free surface available to undergo catalysis again. As a final test of the Lewis acidity of the two materials we examined pyridine adsorption by infra‐red spectroscopy (Figure S29 and Table S18): this showed a strong interaction for UOW‐8 but not for UOW‐7, which is entirely consistent with the superior catalytic properties of UOW‐8, and confirms the importance of Lewis acid sites to enable the reduction of 4‐NP.

## Conclusions

Two novel Fe‐based MOFs, UOW‐7 and UOW‐8 are synthesised using the biomass‐derived ligand, 2,5‐furandicarboxylate (FDC) and structures analysed with 3DED to reveal rare motifs of tetrameric clusters and 1D chains of Fe (III). Although non‐porous, these materials are shown to be effective catalysts in the reduction of 4‐nitrophenol to 4‐aminophenol, and the improved performance of UOW‐8 over UOW‐7 is explained as being due to the availability of Lewis acidic sites at the surface of the particles. The combination of a benign metal with a renewably sourced and environmentally friendly ligand presents these as sustainable MOFs for catalysis and showcases the potential of ligands beyond conventional fossil‐derived options. Our results underscore the strategic design of Fe‐based MOFs for enhanced catalytic properties and pave the way for future research into the sustainable synthesis of MOFs with tailored properties for specific applications, fostering advancements in green chemistry and sustainable materials. Further work is needed to fully establish the catalytic mechanisms at play, and operando experiments and computational approaches will be useful future directions in this respect.

## Conflict of Interests

The authors declare no conflict of interest.

1

## Supporting information

As a service to our authors and readers, this journal provides supporting information supplied by the authors. Such materials are peer reviewed and may be re‐organized for online delivery, but are not copy‐edited or typeset. Technical support issues arising from supporting information (other than missing files) should be addressed to the authors.

Supporting Information

## Data Availability

Deposition numbers 2360468 (for UOW‐7) 2360469 (for UOW‐8) contain the supplementary crystallographic data for this paper. These data can be obtained free of charge from The Cambridge Crystallographic Data Centre via www.ccdc.cam.ac.uk/structures. Other supporting data are available at https://wrap.warwick.ac.uk
