# Supplementary material for: Fe‐2,5‐furandicarboxylate Metal Organic Frameworks with Rare Building Blocks and a Ligand that can be Biomass‐Derived as Effective Catalysts for Selective Nitroarene Reduction
Source: ChemSusChem. 2025 Apr 2;18(11):e202500120. doi: 10.1002/cssc.202500120 (PMC12131673; doi:10.1002/cssc.202500120)
Supplement: Supplementary file 1 — Supporting Information [file CSSC-18-e202500120-s001.pdf]

ChemSusChem

Supporting Information

Fe-2,5-furandicarboxylate Metal Organic Frameworks with Rare Building Blocks and a Ligand that can be Biomass-Derived as Effective Catalysts for Selective Nitroarene Reduction

Satarupa Das, Omar Al-Miqdadi, Wai Wing Cheng, Jeremiah P. Tidey, Marc Walker, Gaurav C. Pandey, and Richard I. Walton*

Fe-2,5-furandicarboxylate Metal Organic Frameworks with Rare Building Blocks and a Ligand that can be Biomass-Derived as Effective Catalysts for Selective Nitroarene Reduction

Satarupa Das,[a] Omar Al-Miqdadi,[b] Wai Wing Cheng,[b] Jeremiah P. Tidey,[b] Marc Walker,[b] Gaurav C. Pandey,[c] Richard I. Walton[a*]

a: Department of Chemistry, University of Warwick, Coventry CV4 7AL, UK

b: Department of Physics, University of Warwick, Coventry CV4 7AL, UK

c: Warwick Manufacturing Group, University of Warwick, Coventry CV4 7AL, UK

*Author for correspondence: r.i.walton@warwick.ac.uk

**Contents**	**Page No.**
Materials	S4
Synthesis of UOW-7 and UOW-8	S4
Catalytic reduction of 4-nitrophenol	S4
Materials characterisation	S5
Structure determination by 3DED	S6
Experimental data table for UOW-7 3DED	S7
Experimental data table for UOW-8 3DED	S8
Lattice parameter comparison from 3DED and PXRD	S9
Kinetic and thermodynamic Analysis	S9-10
XAS analysis	S11-16
IR analysis	S17-18
Thermodiffractometry heatmap for UOW-7	S19
Potential void visualisation in UOW-7	S19
Nitrogen adsorption isotherm measurement of UOW-7	S20
Conformations of Fe_4O_2 cluster	S20
Thermodiffractometry heatmap for UOW-8	S21
Potential void visualisation in UOW-8	S21
Nitrogen adsorption isotherm measurement of UOW-8	S22
Scanning Electron Microscopy images of as-synthesised UOW-7 and UOW-8	S22
X-ray diffraction plot post water stability test of UOW-7 and UOW-8	S23
UV-Vis spectra of bare 4-Nitrophenol for several runs and 4-nitrophenol with addition of $NaBH_4$	S23
UV-Vis spectra for 4-nitrophenol reduction using MlL-53 and corresponding xrd	S24
UV-Vis spectra for 4-nitrophenol reduction using MlL-100 and corresponding xrd	S24
UV-Vis spectra for 4-nitrophenol reduction using Fe_2O_3 + FDCA	S25
Kinetics comparison for UOW-7, UOW-8 and physical mix of Fe_2O_3 + FDCA at RT	S25
Kinetics analysis for MIL-100	S26
Plot of k_{app} vs 4-NP concentration in presence of the catalytic systems	S27
Plot of k_{app} vs $NaBH_4$ concentration in presence of the catalytic systems	S28
Post catalyst powder XRD of UOW-7 and UOW-8	S28
X-ray photoelectron spectra for as synthesised and post catalyst MOFs	S29
Kinetic measurements of UOW-and UOW-8 post 1st and 2nd cycle of catalysis at room temperature	S29
Data obtained from Eyring analysis, linear fits, and calculated rate coefficients for the nitrophenol reduction using UOW-7 catalyst at different temperatures	S30

Data obtained from Eyring analysis, linear fits, and calculated rate coefficients for the nitrophenol reduction using UOW-8 catalyst at different temperatures	S30
Calculated rate coefficients for the nitrophenol reduction using UOW-7, UOW-8 catalyst and Fe_2O_3+ FDCA at RT	S30
Transition state thermodynamic parameters: ΔH≠ and ΔS≠ value	S30
Gibbs free energy of the transition state determined for the catalysts	S31
Comparison chart of catalytic efficiency of UOW-7 and UOW-8 with respect to previously reported iron catalysts	S31
XPS fitting parameters table for UOW-7.	S31
XPS fitting parameters table for UOW-8	S32
Kinetic rate constants post 1st and 2nd cycle of catalysis for UOW-7 and UOW-8 at RT compared against MIL-100	S32
Ir spectroscopy of pyridine adsorption	S33-S34
References	S34-S35

S1: Materials.

The following reagents were purchased from commercial vendors and used as received. NaOH (≥ 98 %, pellets (anhydrous), Sigma Aldrich), furan-2,5-dicarboxylic acid (FDCA, 98%, Alfa Aesar), glacial acetic acid (Fischer Scientific), deionised water, iron chloride hexahydrate ($FeCl_3.6H_2O$, 99%, Merck), iron acetate (95%, Sigma-Aldrich), iron oxide (Fe_2O_3, Alfa Aesar), iron oxide (Fe_3O_4,99.99%, Sigma-Aldrich), p-nitrophenol (99%, Alfa Aesar), sodium borohydride (Sigma-Aldrich)

Synthesis of UOW-7

86 mg (1.26 mmol) of iron acetate was placed into a 23 ml PTFE autoclave liner. Following this, 156 mg (1.5 mmol) of 2,5-furan dicarboxylic acid, 40 mg of NaOH, 4 ml of deionized water, and 1 ml of acetic acid were successively added. The mixture was stirred for 5 minutes, then sealed and heated to 120°C for a period of 24 hours. Upon cooling, the resulting brown powder was gathered via vacuum filtration, washed twice with water and ethanol, and subsequently air-dried at 70°C.

Synthesis of UOW-8

Iron chloride hexahydrate (810 mg,3 mmol) was introduced into a 50 ml round bottom flask. Subsequently, 2,5-furan dicarboxylic acid (468 mg, 3 mmol), 10 ml of deionised water, and 120 mg of sodium hydroxide (4 mmol) were successively added. The reaction mixture was stirred vigorously for 10 minutes. Then the reaction mixture was put under reflux for 24 hrs. post-reaction, the resultant product was collected through vacuum filtration, subjected to two washes with water and two with methanol, and air-dried at 70 °C.

Catalytic reduction of 4-nitrophenol

To catalytically reduce 4-nitrophenol (4-NP), 0.5 mL of a 0.1 M $NaBH_4$ aqueous solution was introduced into a 25 mL aqueous solution of 4-nitrophenol (0.1 mM). Subsequently, 20 mg of MOF catalyst was added. The reduction of 4-nitrophenol to 4-aminophenol (4-AP) was monitored through UV-Vis absorption spectra ranging from 200 to 600 nm.

The conversion percentage of the catalyst is determined by Equation 1:

$$\% \text{ Conversion} = \frac{Co - Ct}{Co} \times 100\%$$

Here, C_0 is the initial concentration (corresponding to absorbance) of 4-nitrophenol at λ_{max}, and C_t is the concentration at time t.

In the recycle test, 5 cycles of activity were measured under the identical conditions.

S2: Materials Characterisation

Powder X-ray diffraction (PXRD) patterns for various samples were acquired using a Siemens D5000 diffractometer equipped with Cu K$\alpha_{1/2}$ radiation, employing Bragg-Brentano mode with a step size of $\Delta 2\theta = 0.02°$ and a recording time of 4 seconds per step. Synchrotron PXRD experiments were conducted at the Diamond Light Source in the United Kingdom, utilizing the high-resolution beamline I11. The diffraction patterns were captured in transmission mode over the range of $[0° < 2\theta < 150°]$, employing a position-sensitive detector (PSD) with a wavelength (λ) of 0.825379 Å. These measurements were carried out at room temperature, with samples contained within borosilicate glass capillaries of 0.7 mm diameter. The catalyst's morphology was examined through a Zeiss Supra 55-VP field emission scanning electron microscope (FESEM). Thermogravimetric analysis (TGA) was conducted on a Mettler Toledo TGA/DSC1 instrument under ambient air pressure, employing a heating rate of 10 °C per minute, with the samples heated from 25 °C to 1000 °C. Inductively coupled plasma (ICP) spectroscopy for chemical catalyst analysis was carried out using a Varian Vista MPX ICP-OES system by Medac Limited. The UV-VIS spectroscopy data were obtained using Cary60 UV/VIS instrument. was utilized to calculate the apparent surface area. The X-ray photoelectron spectroscopy (XPS) data were collected at the Photoemission RTP, University of Warwick. The samples investigated in this study were attached to electrically conductive carbon tape, mounted on to a sample bar with a layer of filter paper between the samples and the sample bar to ensure electrical isolation, before being loaded into a Kratos Axis Ultra DLD spectrometer which possesses a base pressure below 1 x 10^{-10} mbar. The XPS measurements were performed in the main analysis chamber, with the sample being illuminated using a monochromated Al Kα x-ray source ($h\nu = 1486.7$ eV). The measurements were conducted at room temperature and at a take-off angle of 90° with respect to the surface parallel. The core level spectra were recorded using a pass energy of 20 eV (resolution approx. 0.4 eV), from an analysis area of 300 x 700 mm. The work function and binding energy scale of the spectrometer were calibrated using the Fermi edge and $3d_{5/2}$ peak recorded from a polycrystalline Ag sample prior to the commencement of the experiments. To prevent surface charging the surface was flooded with a beam of low energy electrons from a charge neutraliser throughout the experiment and this necessitated recalibration of the binding energy scale. To achieve this, the C-C/C-H component in the C 1*s* spectrum was referenced to 285.0 eV. The data were analysed in the CasaXPS package using Shirley backgrounds and mixed Gaussian-Lorentzian (Voigt) lineshapes. For compositional analysis, the analyser transmission function has been determined using clean metallic foils to determine the detection efficiency across the full binding energy range. The X-ray absorption measurements were carried out using an easyXAFS300+ instrument[1] at the Fe K-edge. Samples were diluted with cellulose powder and pressed into 1 mm thick pellets, to optimise absorption measurements and data recorded in transmission mode. The raw XAFS data were processed using the software ATHENA[2] for background removal (both pre- and post-edges), absorption energy (E_0), and normalisation. The E_0 was determined as the half height of the normalised absorption edge. The EXAFS spectra were analysed using the software ARTEMIS[2] over a k-range of 3-10.5 Å^{-1}, using structure models based on the crystal structures of the materials fitted to the k^3-weighted spectrum, and the FEFF code for the calculation of phase shifts and effective scattering amplitudes with single scattering paths used

in the fitting. FT-IR spectra were recorded using a Bruker ALPHA FTIR ATR spectrometer with measurements carried out on solid powder samples in transmission mode. For pyridine adsorption studies a Cary 630 FTIR Spectrometer was used with pyridine added to freshly dried solid and allowed to evaporate to leave behind the pyridine-adsorbed sample, while repeated scans were made. XRF was carried out using a Rigaku PrimusIV wavelength-dispersive X-ray fluorescence spectrometer (WDXRF)equipped with a 4 kW X-ray tube.

S3: Structure determination

For the 3DED experiment, samples were lightly ground between two glass slides and dispersed onto a copper-supported continuous carbon TEM grids and loaded via a Gatan Elsa model 689 cryo holder into a Rigaku XtaLAB Synergy-ED electron diffractometer, operated at 200 kV and equipped with a Rigaku HyPix-ED hybrid pixel array area detector.

In each case, data for a range of crystallites were surveyed at 150 K using continuous rotation electron diffraction with a selected area of ~2 μm diameter at the image plane using CrysAlisPRO (version 1.171.43.74a for UOW-7 and 1.171.43.80a for UOW-8 (Rigaku Oxford Diffraction, 2025)). For UOW-7, the reported data are taken from four representative datasets of comparable quality from ~1 μm termini of crystallites appearing as slabs ~1×0.5×0.25 μm in size and, for UOW-8, three representative datasets were taken from crystallites appearing as striated rods of ~3×1×0.25 μm. Individual datasets were taken over tilt ranges of around 105±10 ° using frame scan widths of 0.25 °. Datasets were in each case individually indexed and integrated, and subsequently merged and scaled together for each sample using CrysAlisPRO (version 1.171.44.91a (Rigaku Oxford Diffraction, 2025)) and SCALE3 ABSPACK, implemented therein.

The structures were solved using ShelXT [3] and refined in the kinematic approximation using Olex2.refine[4] as implemented in Olex2, version 1.5-ac7-013 (compiled 2025.01.02 svn.rf662f148 for Rigaku Oxford Diffraction, GUI svn.r7109[5]) using published scattering factors [6]. An extinction correction was applied in each case to broadly account for the impact of multiple diffraction with further omission of particularly outlying reflections in the final stages of the refinement, treated as either significantly affected by multiple scattering, or either being in some way obscured during collection or by limitations in the automated outlier rejections otherwise used.

In all cases, C-H hydrogen atoms were placed at geometrically constrained positions at neutron distances with riding isotropic displacement parameters. Not all water protons could be located stably and are omitted where this was the case. Those which were able to be stably refined (solely in UOW-8) were located in the difference map and refined in the presence of 1,2 and 1,3 distance similarity restraints. In both samples, rigid bond restraints were applied to better model anisotropic displacement parameters for non-hydrogen atoms, while hydrogen atoms were treated with riding isotropic displacement parameters.Complete experimental and refinement information are contained in the deposited CIFs along with structure factors and embedded .RES files. These are deposited in the CSD with CCDC reference codes CCDC 2360468-2360469. Tables 1 and 2 report experimental parameters from the merged refinement and individual datasets, respectively.

Table S1. Experimental data table for UOW-7 3DED.

**Merged Crystal Data**				
Chemical formula	$C_{28}H_{14}Fe_5NaO_{27}$·O			
M_r	1100.61			
Crystal system, space group	Orthorhombic, *Cmce*			
Temperature (K)	150			
a, *b*, *c* (Å)	21.0996(15), 17.9635(14), 21.425(4)			
V (Å^3)	8120.4(16)			
Z	4			
Radiation type	Electron, λ = 0.02510 Å			
No. Crystals	4			
**Distinct Data Collections**				
a (Å)	21.096(2)	21.114(4)	21.029(3)	21.177(3)
b (Å)	17.936(3)	17.974(6)	18.010(2)	17.934(2)
c (Å)	21.470(13)	21.393(5)	21.486(12)	21.404(10)
V (Å^3)	8124(5)	8119(4)	8137(5)	8129(4)
R_{int}	0.3454	0.3022	0.2998	0.2878
h (min, max)	-25, 25	-26, 26	-26, 26	-26, 26
k (min, max)	-22, 22	-18, 18	-22, 22	-22, 22
l (min, max)	-21, 21	-24, 25	-23, 23	-21, 22
**Merged dataset**				
No. reflections (meas., indep., obs [I ≥ 2u(I)])	60491, 3358, 2756			
R_{int}	0.349			
$(\sin \theta/\lambda)_{max}$ (Å^{-1})	0.598			
h (min, max)	0, 25			
k (min, max)	0, 21			
l (min, max)	0, 23			
**Refinement**				
R_1, wR_2 [$F^2 > 2\sigma(F^2)$]	0.1913, 0.4431			
R_1, wR_2, GoF(*S*) [all]	0.2122, 0.4521, 1.6173			
No. of reflections	3358			
No. of parameters	289			
No. of restraints	267			
$\Delta\varphi_{max}$, $\Delta\varphi_{min}$ (as output by Olex2.refine)	1.19, −0.74			

Table S2. Experimental data table for UOW-8 3DED.

**Merged Crystal Data**			
Chemical formula	$C_{12}H_8Fe_2O_{13}\cdot4(H_2O)$		
M_r	543.93		
Crystal system, space group	Monoclinic, *C*2/*c*		
Temperature (K)	150		
a, *b*, *c* (Å)	20.101 (6), 10.6810 (8), 20.255 (4)		
β (°)	119.43(3)		
V (Å^3)	3787.6(19)		
Z	8		
Radiation type	Electron, λ = 0.02510 Å		
No. Crystals	3		
**Distinct Data Collections**			
a (Å)	20.07(2)	20.129(8)	20.067(17)
b (Å)	10.6807(13)	10.6853(16)	10.7006(15)
c (Å)	20.280(10)	20.219(9)	20.257(8)
β (°)	119.40(9)	119.31(5)	119.46(7)
V (Å^3)	3787(4)	3792(2)	3787(3)
R_{int}	0.1516	0.1792	0.1835
h (min, max)	-18, 19	-25, 25	-22, 22
k (min, max)	-13, 13	-13, 13	-11, 12
l (min, max)	-25, 25	-21, 21	-25, 25
**Merged dataset**			
No. reflections (meas., indep., obs [I ≥ 2u(I)])	23691, 3796, 2844		
R_{int}	0.215		
$(\sin \theta/\lambda)_{max}$ (Å^{-1})	0.626		
h (min, max)	-21, 25		
k (min, max)	0, 13		
l (min, max)	0, 25		
**Refinement**			
R_1, wR_2 [$F^2 > 2\sigma(F^2)$]	0.166, 0.3273		
R_1, wR_2, GoF(*S*) [all]	0.2019, 0.3473, 1.0058		
No. of reflections	3796		
No. of parameters	318		
No. of restraints	300		
$\Delta\varphi_{max}$, $\Delta\varphi_{min}$ (as output by Olex2.refine)	1.40, −1.48		

Table S3: Lattice parameters of UOW-7 from the 3DED single-crystal structure determination and Pawley refined against powder XRD patterns.

**Lattice Parameters**	**Single Crystal (150 K)**	**Pawley refinement (293 K)**
a/Å	21.12(13)	21.35 (3)
b/Å	17.91(12)	18.163052(5)
c/Å	21.5(3)	21.722864(3)
V/Å3	8130(120)	8424.621(3)
*R*wp / %	-	9.63

Table S4: Lattice parameters of UOW-8 from the 3DED structure determination and Pawley refined against powder XRD patterns.

**Lattice Parameters**	**Single Crystal (150 K)**	**Pawley refinement (293 K)**
a/Å	20.1(3)	20.071(3)
b/Å	10.69(8)	10.7044(6)
c/Å	20.25(16)	20.182(4)
V/Å3	3790(90)	3790.8(2)
b/°	119.5(16)	119.041(6)
*R*wp / %	-	3.880

S4: Kinetic and thermodynamic analysis of catalysis

The analysis of the kinetic data was done in terms of the Langmuir-Hinshelwood model. In the Langmuir-Hinshelwood (LH) model, the apparent kinetic rate constant, k_{app}, exhibits a strict proportionality to the total surface (S) encompassing all catalyst active sites. The kinetic rate constants k_{app} and k_1 within this model can be precisely defined as follows:

$$\frac{dc_{NP}}{dt} = -k_{app} \times c_{NP} = -k_1 \times S \times c_{NP} \quad (1)$$

The LH model further implies,

$$\frac{dc_{NP}}{dt} = -k \times S \times \theta_{NP} \times \theta_{BH4} \quad (2)$$

The surface coverage of the catalyst by 4-nitrophenol and borohydride is represented by θ_{NP} *and* θ_{BH4}, respectively, while k denotes the rate constant of the surface reaction. These parameters are subject to modelling through a Langmuir isotherm.

$$\theta_{NP} = \frac{(K_{NP}c_{NP})^n}{1+ (K_{NP}c_{NP})^n + K_{BH4}c_{BH4}} \tag{3}$$

$$\theta_{BH4} = \frac{(K_{BH4}c_{BH4})^n}{1+ (K_{NP}c_{NP})^n + K_{BH4}c_{BH4}} \tag{4}$$

$$-\frac{dc_{NP}}{dt} = \frac{kS(K_{NP}c_{NP})^n(K_{BH4}c_{BH4})^m}{\left(1 + (K_{NP}c_{NP})^n + (K_{BH4}c_{BH4})^m\right)^2} = k_{app} \times c_{NP} \tag{5}$$

Therefore,

$$k_{app} = \frac{kS\, K_{NP}c_{NP}{}^n(K_{BH4}c_{BH4})^m}{\left(1 + (K_{NP}c_{NP})^n + (K_{BH4}c_{BH4})^m\right)^2} = c_{NP} \tag{6}$$

In this context, K_{Np} and K_{BH4} represent the adsorption constants for Np and BH_4^-, respectively, while c_{Np} and c_{BH4} denote their respective concentrations in the solution. The exponent n is associated with the heterogeneity of the sorbent. The Langmuir-Freundlich equation considers adsorption energy is not same across different sites, depicting it as a Gaussian distribution. A broader distribution signifies greater surface heterogeneity, resulting in a smaller value for the exponent. Hence, case of $n = 1$ represents the classical Langmuir isotherm in which all sites have the same adsorption energy. With this modification, equation 1, can be written as

$$k_{app} = \frac{kSK_{NP}^n c_{NP}^{n-1}(K_{BH4}c_{BH4})^m}{\left(1 + (K_{NP}c_{NP})^n + (K_{BH4}c_{BH4})^m\right)^2} = c_{NP} \tag{7}$$

The ln([*A*]$_t$/[*A*]0) versus time plot underwent fitting using first-order kinetics (as per Equation 2) to ascertain the reaction's rate constant (k). The high goodness-of-fit ($R^2 \sim 1$) of the linear regression implies that the reaction indeed adheres to pseudo-first-order kinetics.

$$ln[A]_t = ln[A]_0 - kt \tag{8}$$

Additionally, the determined rate constants at different temperatures (0, 25, and 60 ºC) were utilized to ascertain the thermodynamic parameters, specifically the activation enthalpy ($\Delta H^{\neq}$) and activation entropy ($\Delta S^{\neq}$), utilizing the Eyring equation (Equation 3). In this equation, *k* represents the rate constant, *T* denotes temperature, *R* is the universal gas constant (8.314 J K^{-1} mol^{-1}), k_B is the Boltzmann constant (1.38×10^{-23} m^2 kg s^{-2} K^{-1}), and *h* is the Planck constant (6.626×10^{-34} m^2 kg s^{-1}).

$$ln\left(\frac{k}{T}\right) = -\left(\frac{\Delta H^{\neq}}{R}\right)\cdot\left(\frac{1}{T}\right) + \left(\frac{\Delta S^{\neq}}{R} + ln\left(\frac{k_B}{h}\right)\right) \tag{9}$$

The determined ($\Delta H^{\neq}$) and ($\Delta S^{\neq}$) from the slope and intercept of the ln(*k*/*T*) versus (1/*T*) plot were further employed to calculate the Gibbs free energy of the reaction with different MOF catalysts, as per Equation (4):

$$\Delta G^{\neq} = \Delta H^{\neq} - T\Delta S^{\neq} \tag{10}$$

S5: XAFS Analysis

α-Fe_2O_3 reference material

The Fe K-edge EXAFS can be fitted with several shells of atoms that match the expected crystal structure of α-Fe_2O_3. Beyond the local coordination of six oxygens, three further shells of Fe can be seen, and intermediate shells of oxygen could not be fitted.

Table S5: Fitted EXAFS parameter for crystalline α-Fe_2O_3. R_{cryst} is the expected interatomic distance from the crystal structure

Shell	R / Å	R_{cryst} / Å	$σ^2$ / Å^2
6 O	1.988(21)	2.029	0.013(4)
4 Fe	2.942(19)	2.950	0.006(2)
3 Fe	3.370(27)	3.362	0.003(3)
3O	-	3.397	-
3O	-	3.592	-
6 Fe	3.674(29)	3.702	0.009(4)

S_0^2 (amplitude reduction factor) = 0.869, E_0 = -0.480 eV; R = 0.022

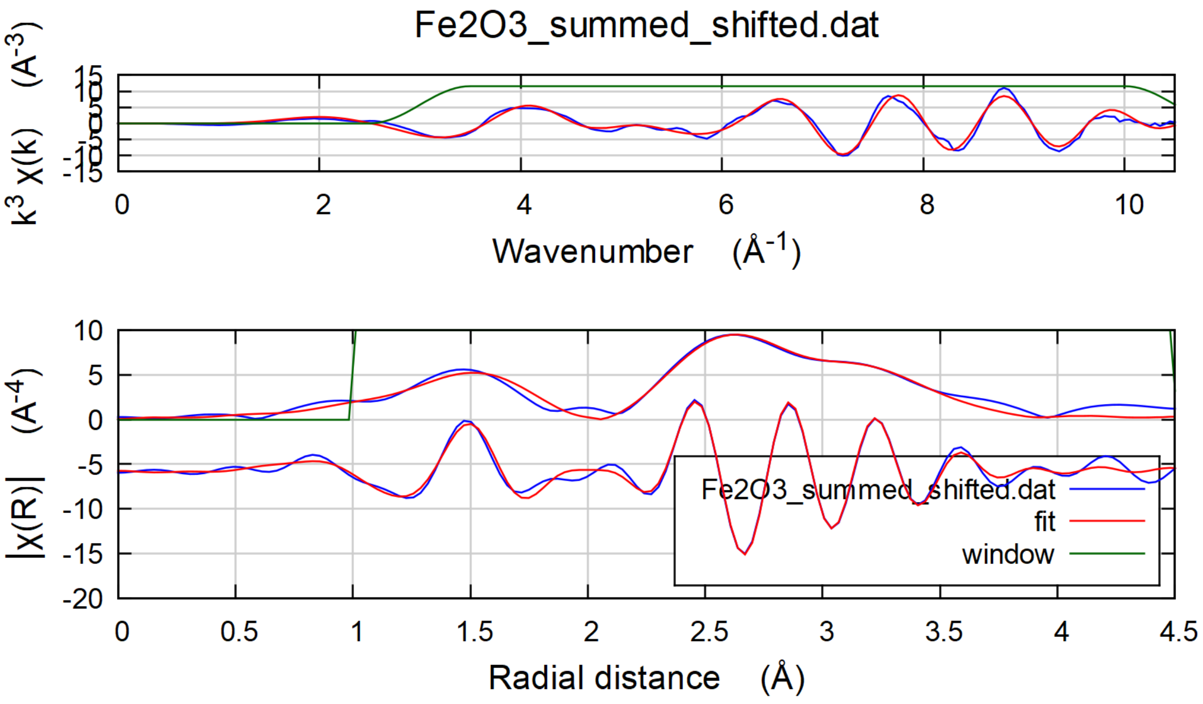

Figure S1: Top: k^3-weighted EXAFS fit for_α-Fe_2O_3. Bottom: Corresponding fit of radial distribution with real part of $χ(R)$ as well as $|χ(R)|$. Blue is the measured function and red is the calculated from final fitted model.

UOW-7

The infinite chain of connected FeO_6 octahedra contains 3 crystallographically distinct Fe centres. This gives each Fe 6 oxygen near neighbours (~2 Å) and 4 second nearest Fe centres over range 2.9 – 3.5 Å (average 3.5 Å). Each Fe is ~ 3 Å from 4 or 6 carboxylate carbons (average 4.67). No evidence for shells of higher *r* is seen in the EXAFS spectrum. Including these 3 shells gave a satisfactory with S_0^2 remaining close to 1.

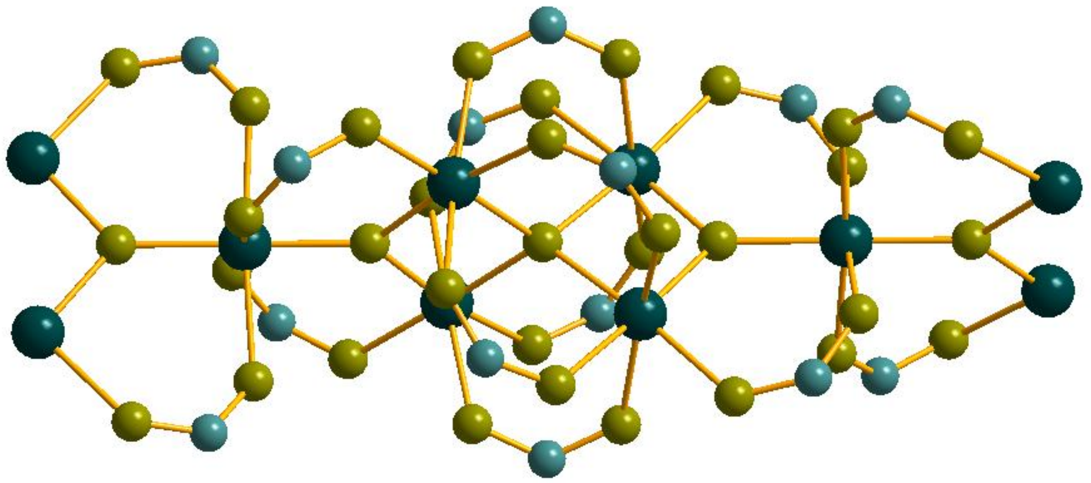

Figure S2: Fragment of UOW-7 structure showing local atomic environment

Table S6: Fitted EXAFS parameter for crystalline UOW-7. *R*cryst is the expected interatomic distance from the crystal structure

Shell	*R* / Å	*R*cryst / Å	s² / Å²
6 O	2.026(22)	2.002	0.012(4)
4.67 C	2.982(49)	3.096	0.009(7)
4 Fe	3.424(40)	3.350	0.014(5)

S_0^2 = 1.184, E_0 = 4.623 eV; *R* = 0.053

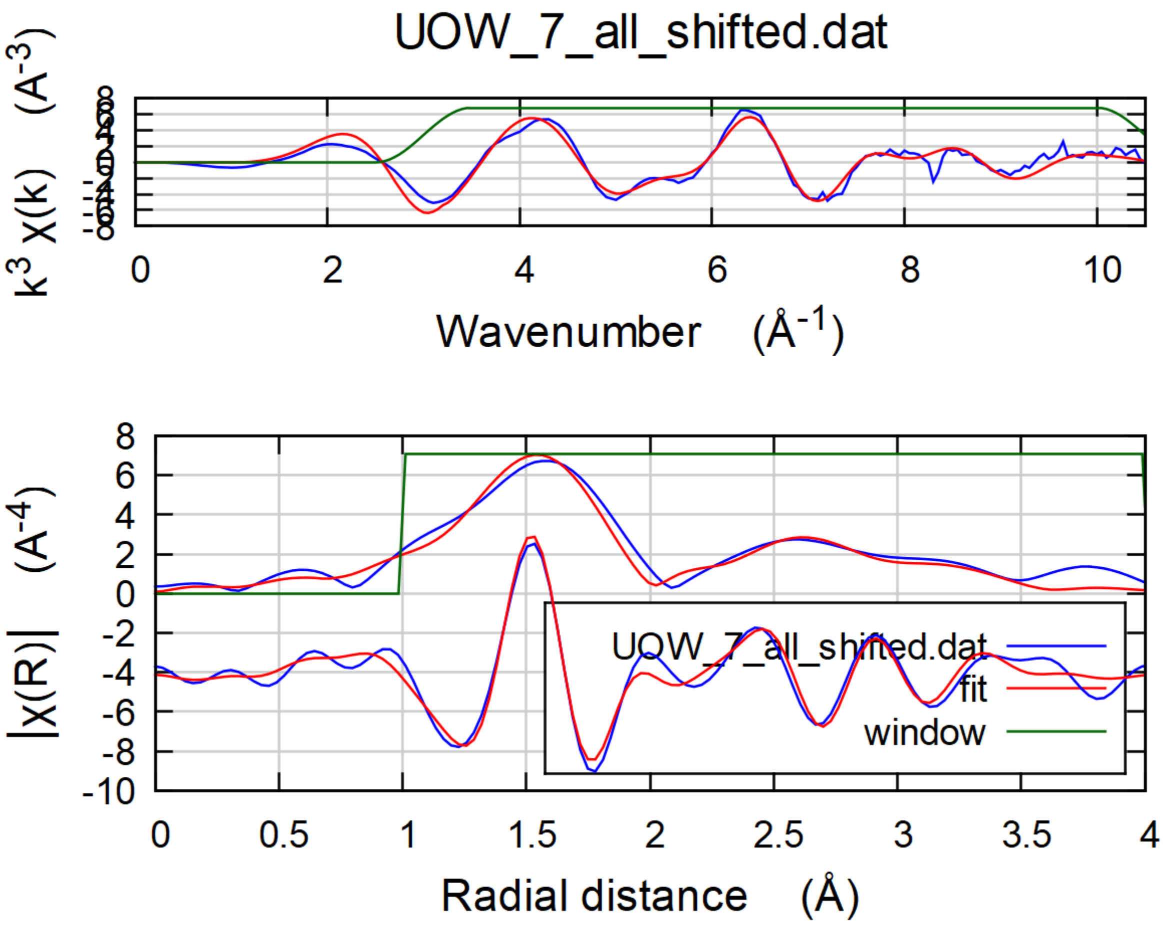

Figure S3: Top: k^3-weighted EXAFS fit for UOW-7. Bottom: Corresponding fit of radial distribution with real part of $\chi(R)$ as well as $|\chi(R)|$. Blue is the measured function and red is the calculated from final fitted model.

UOW-8

The tetrameric building unit contains 3 crystallographically distinct Fe centres, each octahedrally coordinated by 6 oxygen atoms (2.0 Å). Two of the Fe in the cluster have 2Fe neighbours, while the other two have 3 Fe neighbours, so that on average each Fe has 2.5 Fe neighbours at a distance of 3.290 Å. Each Fe has a shell of carbons from carboxylates at ~3.1 Å, with an average of 4 C neighbours.

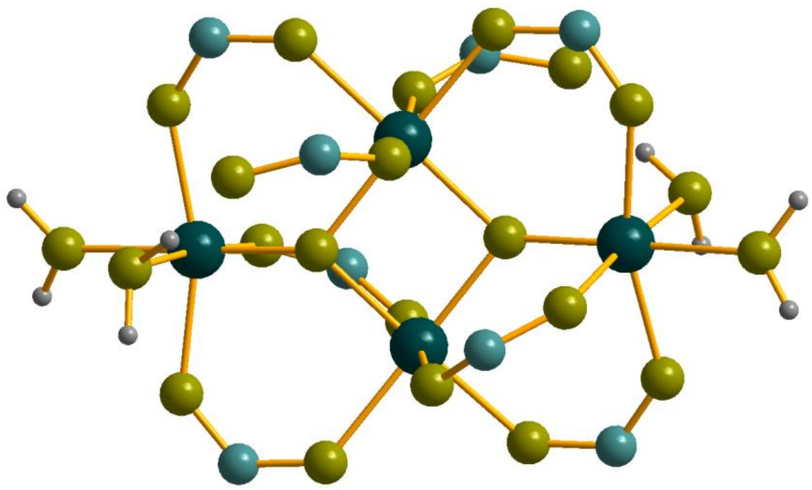

Figure S4: Fragment of UOW-8 structure showing local atomic environment

Table S7: Fitted EXAFS parameter for crystalline UOW-8. *R*cryst is the expected interatomic distance from the crystal structure

**Shell**	***R* / Å**	***R*cryst / Å**	**s² / Å²**
6 O	2.015(21)	2.000	0.013(4)
4 C	2.968(38)	3.110	0.006(5)
2.5 Fe	3.357(32)	3.250	0.010(4)

$S_0^2 = 1.294$ $E_0 = 4.057$ eV; $R = 0.0448$

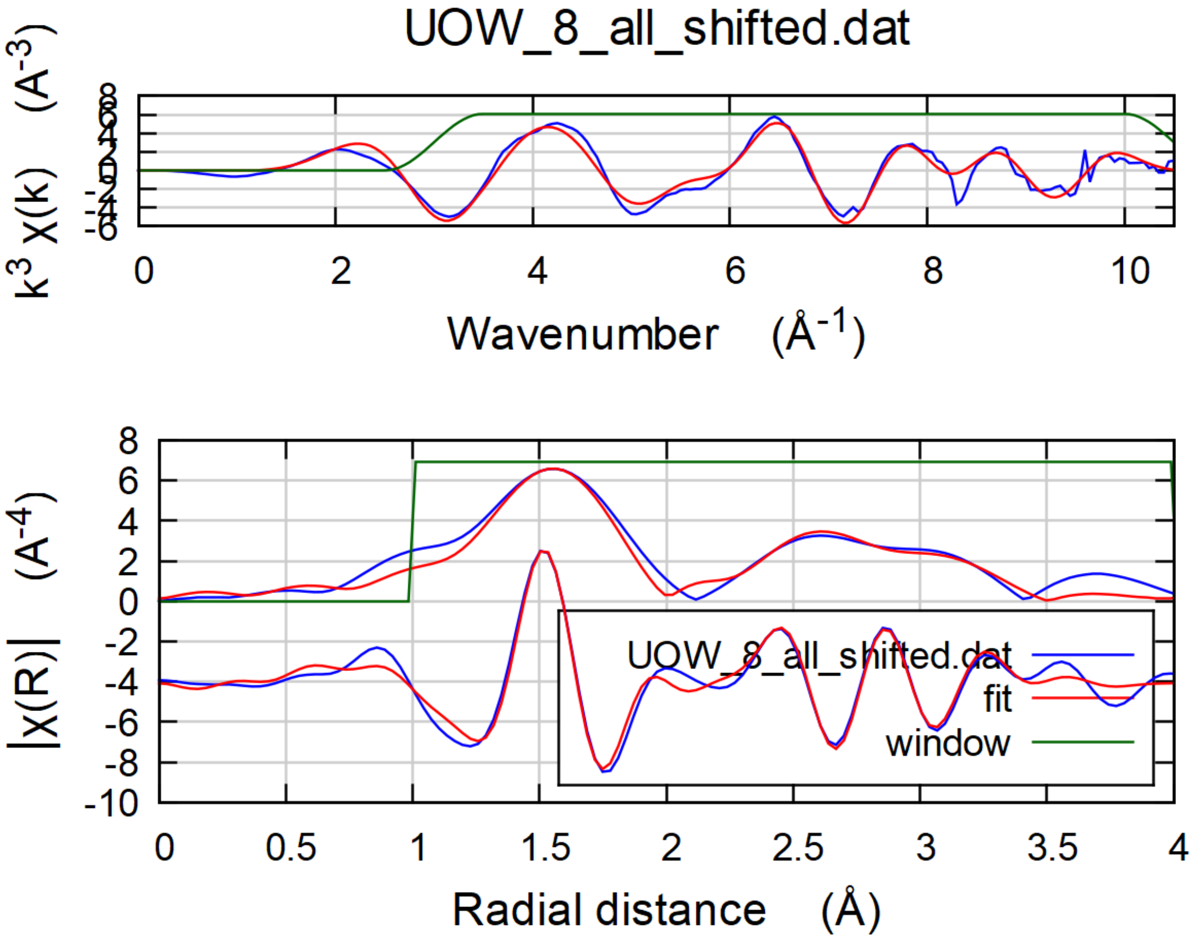

Figure S5: Top: k^3-weighted EXAFS fit for UOW-8. Bottom: Corresponding fit of radial distribution with real part of $\chi(R)$ as well as $|\chi(R)|$. Blue is the measured function and red is the calculated from final fitted model.

Fe K-edge XANES and bond valence sum analysis of crystal structure

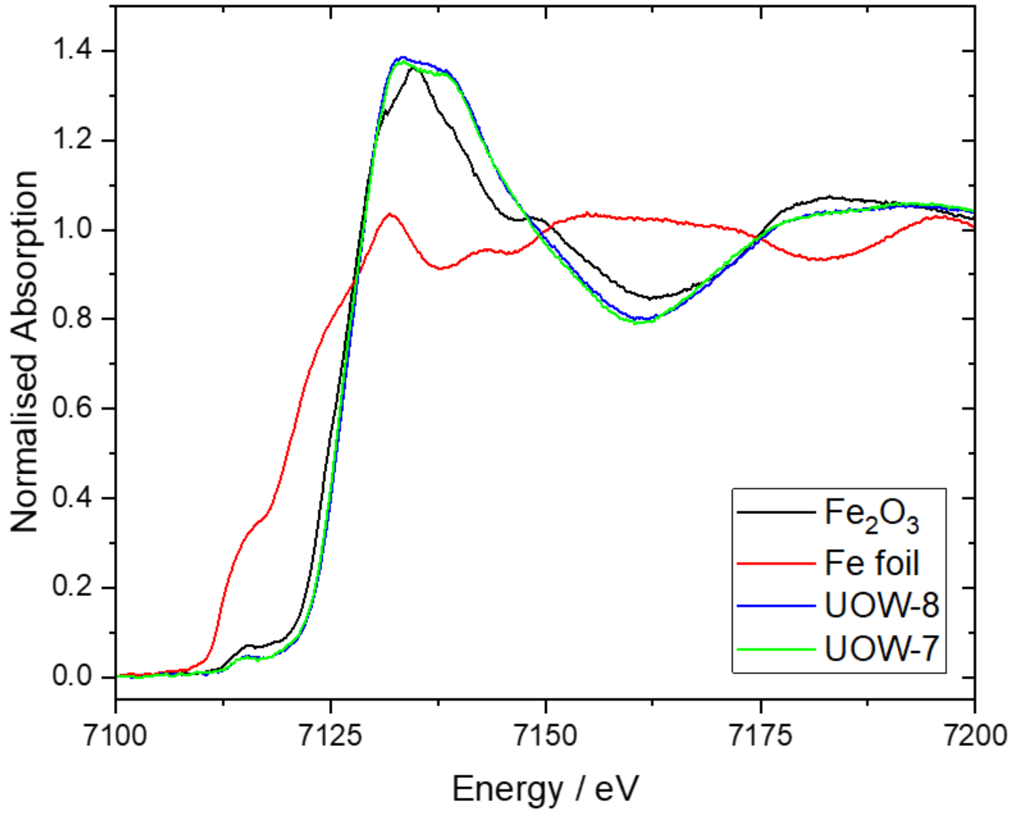

Figure S6: Fe K-edge XANES spectra

The two MOF materials show a similar edge shift from metallic Fe as does Fe_2O_3, consistent with the expected Fe(III) oxidation state.

Bond valence sums for the two materials were carried our using the crystallographic distances and the parameters of Brese and O'Keeffe.[7]

Table S8: Bond valence sum analysis for Fe in UOW-7 and UOW-8

Material	Iron label	BVS
**UOW-7**	Fe0	3.02
	Fe1	3.27
	Fe2	3.20
**UOW-8**	Fe1	3.12
	Fe2	3.23
	Fe3	3.23

S6: Infra-red spectroscopy

The carboxylate region of the IR spectra, Figure S1, show the expected symmetric and asymmetric vibrations for both materials. The bands of UOW-8 are shifted to lower wavenumber and show a more complex structure, which is consistent with the uncoordinated carboxylate oxygen, and unsymmetrical conformation of in one of the ligands (Type 3, see Table S7).

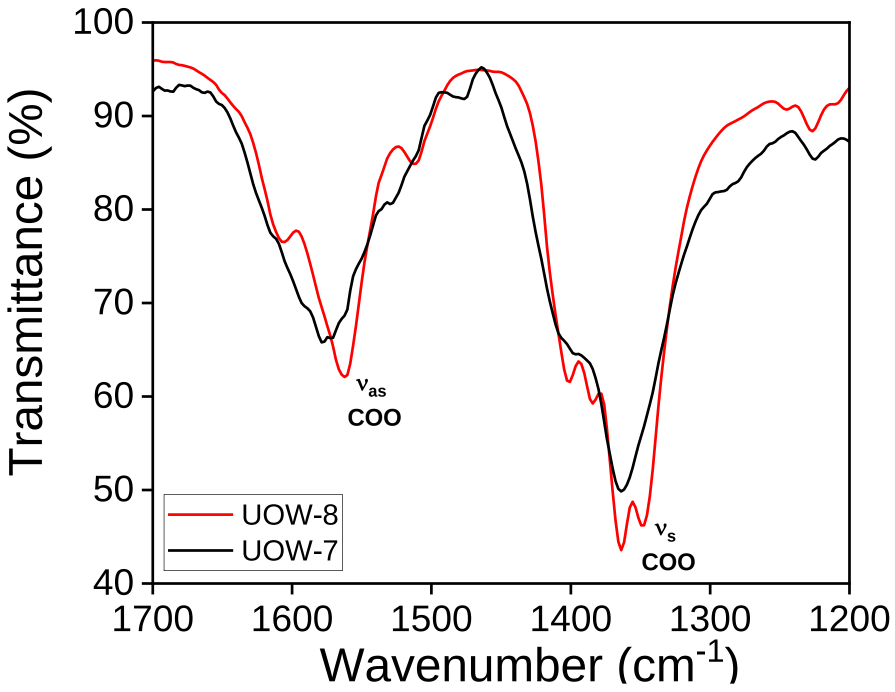

Figure S7: Carboxylate region of the IR spectra of UOW-7 and UOW-8

Table S9: Binding modes of FDC in UOW-7 and UOW-8

**UOW-7**	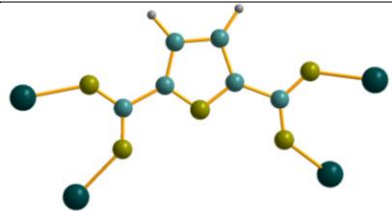	Type 1<br>*Z,Z*-μ_2-η^1: η^1 ; *Z,Z*-μ_2-η^1: η^1
	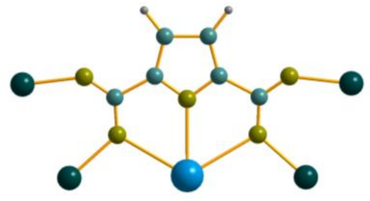	Type 2<br>μ_3-η^1: η^2 ; μ_3-η^1: η^2
**UOW-8**	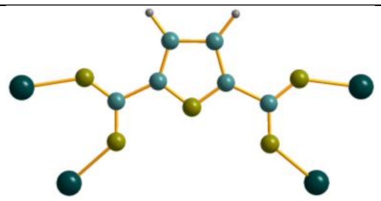	Type 1<br>*Z,Z*-μ_2-η^1: η^1 ; *Z,Z*-μ_2-η^1: η^1

	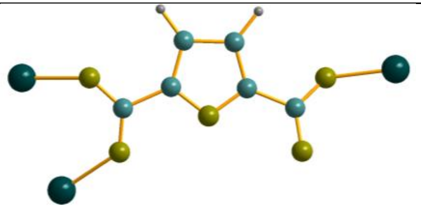	Type 3<br>Z,Z-μ_2-η^1: η^1 ; η^1

The O-H stretch region of the IR spectra confirms the presence of the bound water in UOW-8, and the absence of ordered crystal water in UOW-7 (Figure S8)

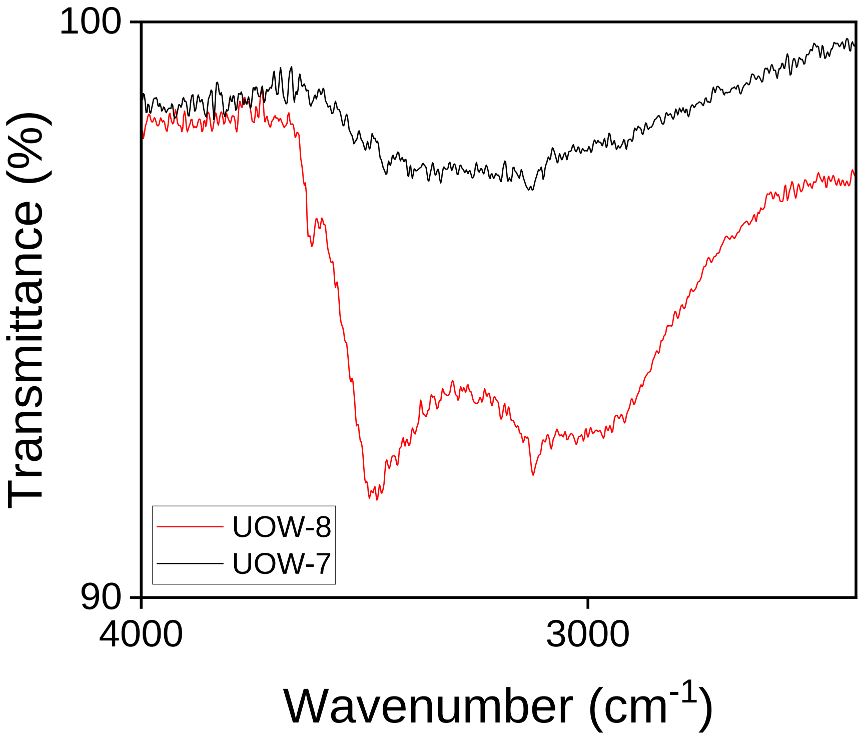

Figure S8: O-H stretch region of the IR spectra of UOW-7 and UOW-8

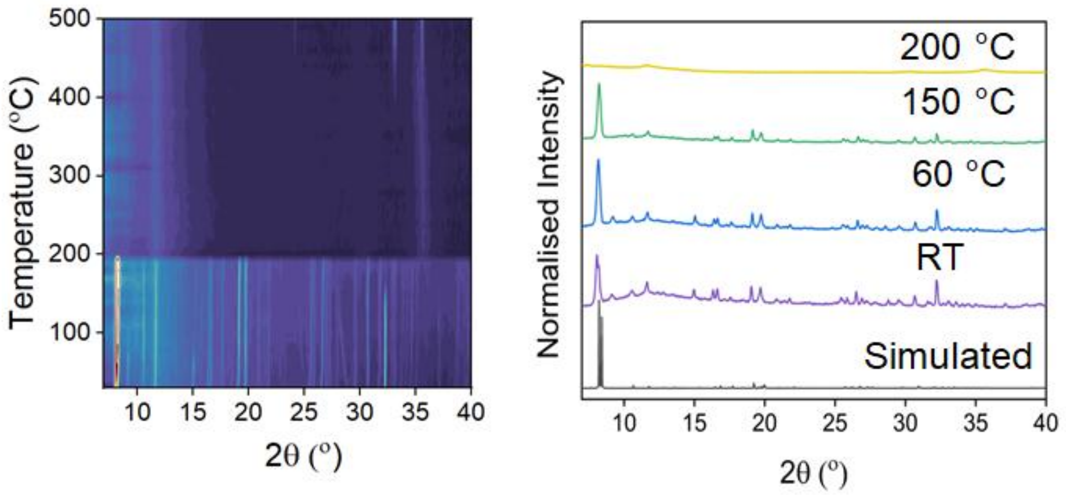

Figure S9. Thermodiffractometry heatmap for UOW-7 and the corresponding XRD at different heating stage compared against the simulated XRD pattern

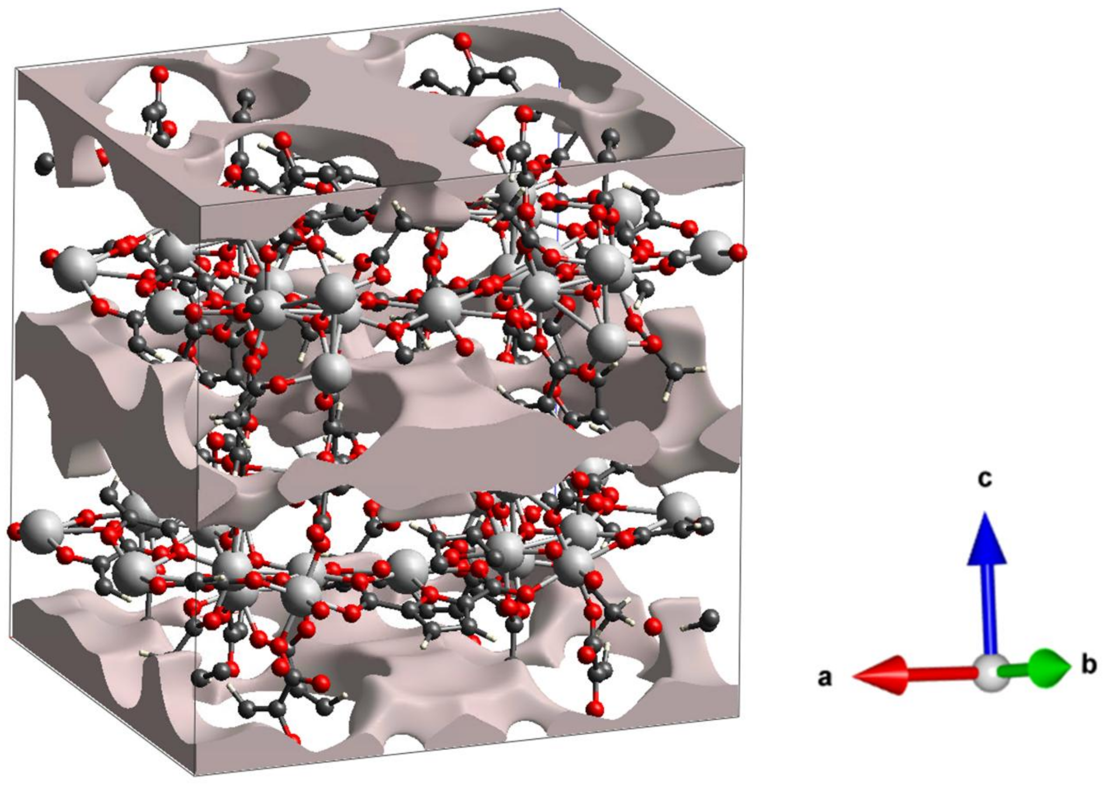

Figure S10. Potential Void visualisation in UOW-7. The calculation was done using CrystalViewer software with an iso value of 0.0003 e/au.

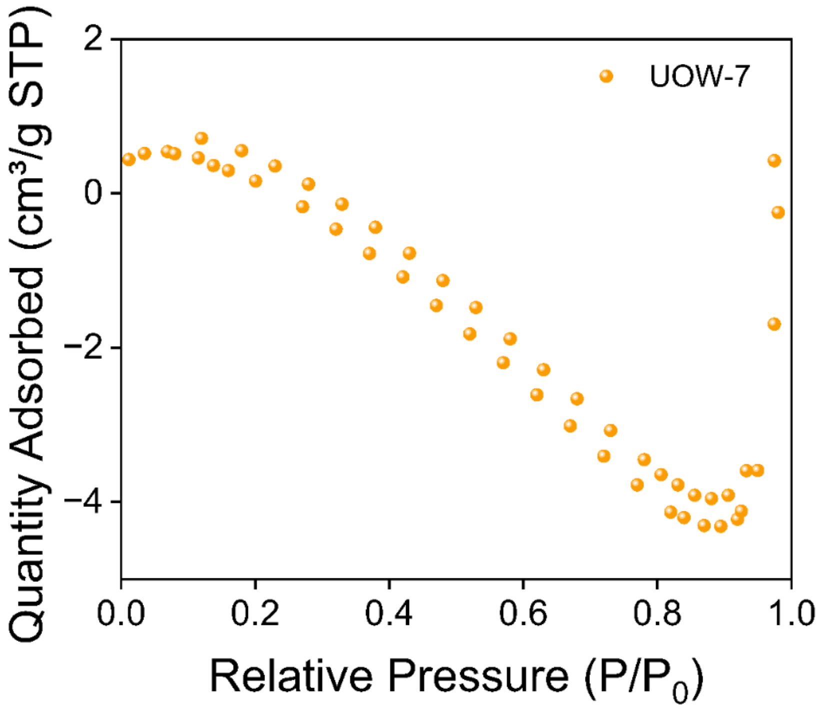

Figure S11. Nitrogen adsorption isotherm measurement of UOW-7 showing that no measurable porosity could be determined.

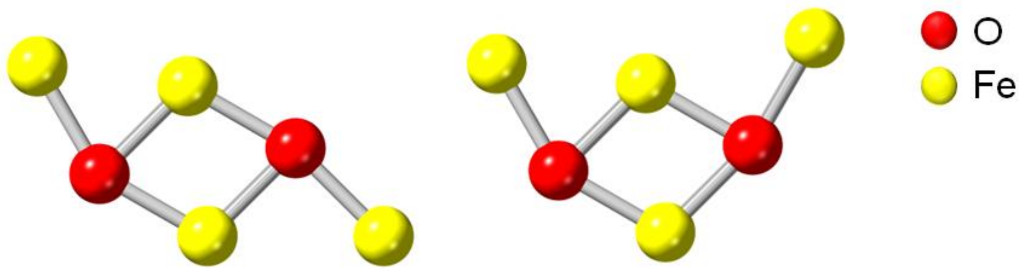

Figure S12. Two distinct possible conformations of Fe_4O_2 cluster

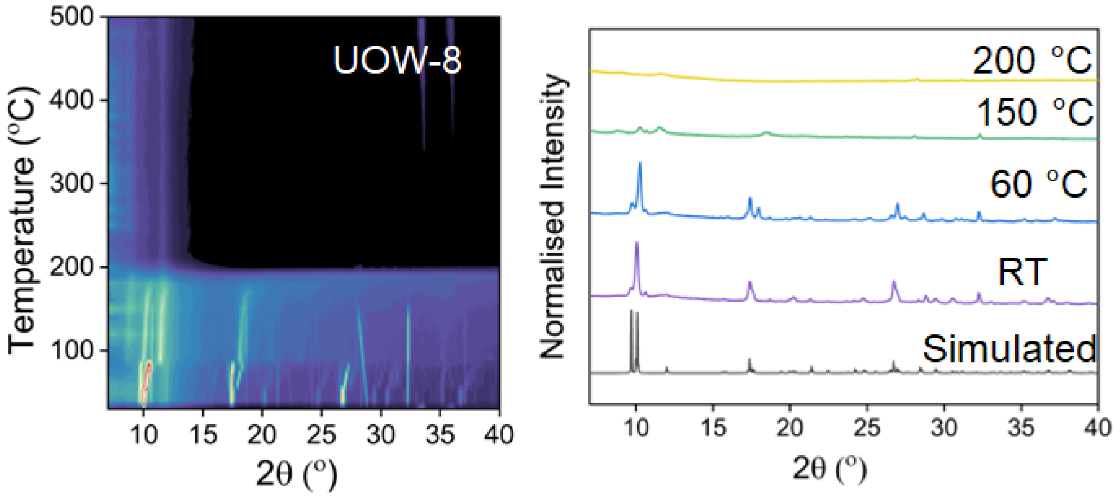

Figure S13. Thermodiffractometry heatmap for UOW-8 and the corresponding XRD at different heating stage compared against the simulated XRD pattern

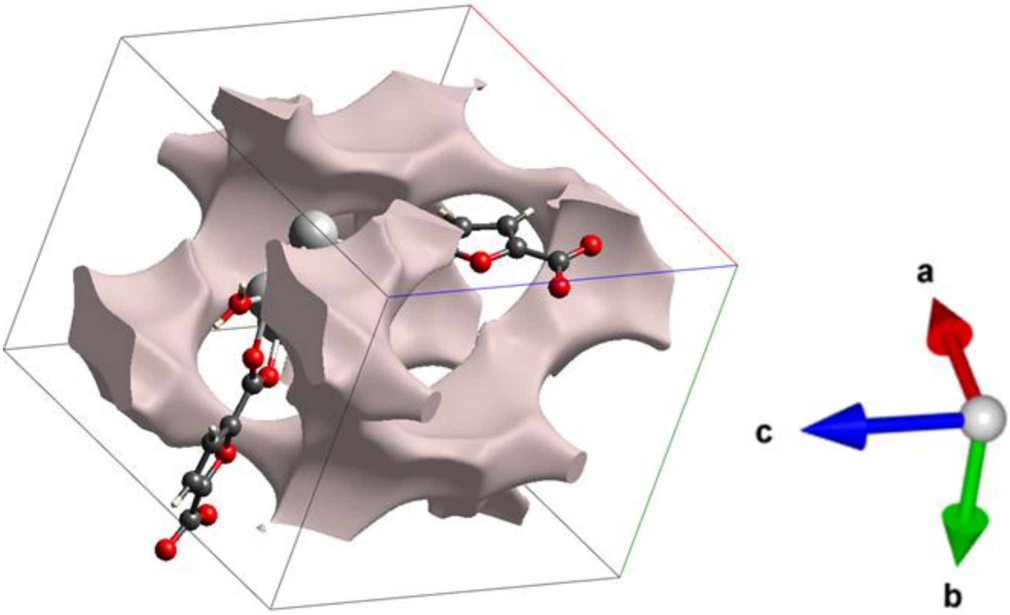

Figure S14. Potential Void visualisation in UOW-8. The calculation was done using CrystalViewer software with an iso value of 0.0003 e/au.

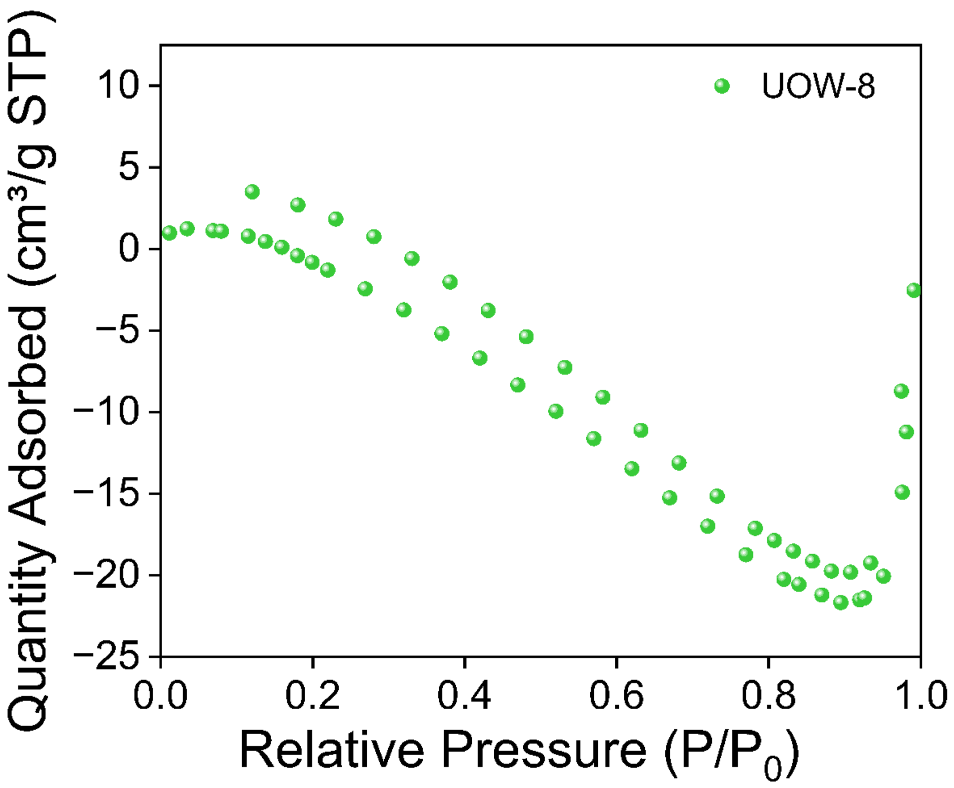

Figure S15. Nitrogen adsorption isotherm measurement of UOW-8 showing that no measurable porosity could be determined.

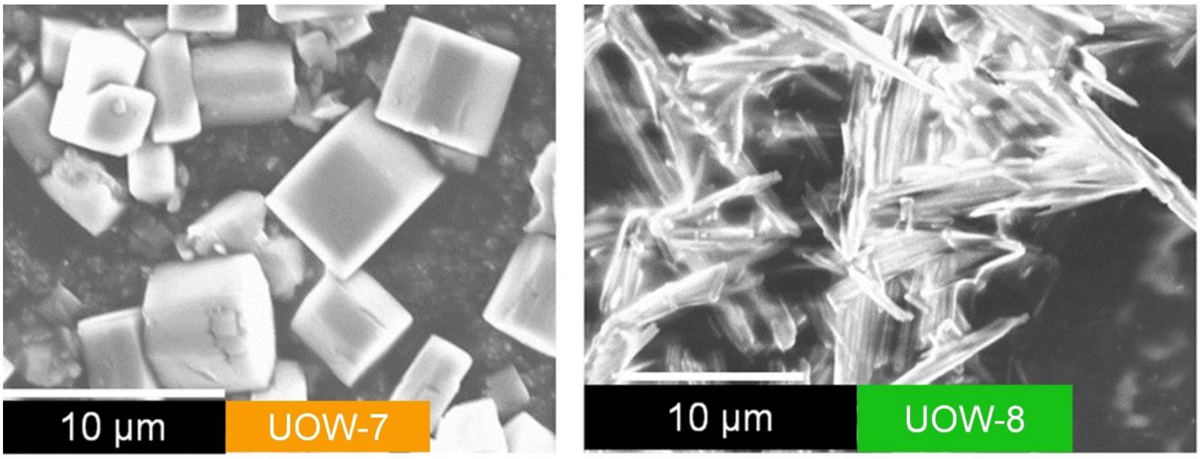

Figure S16. Scanning electron microscopy images of as-synthesised UOW-7 and UOW-8

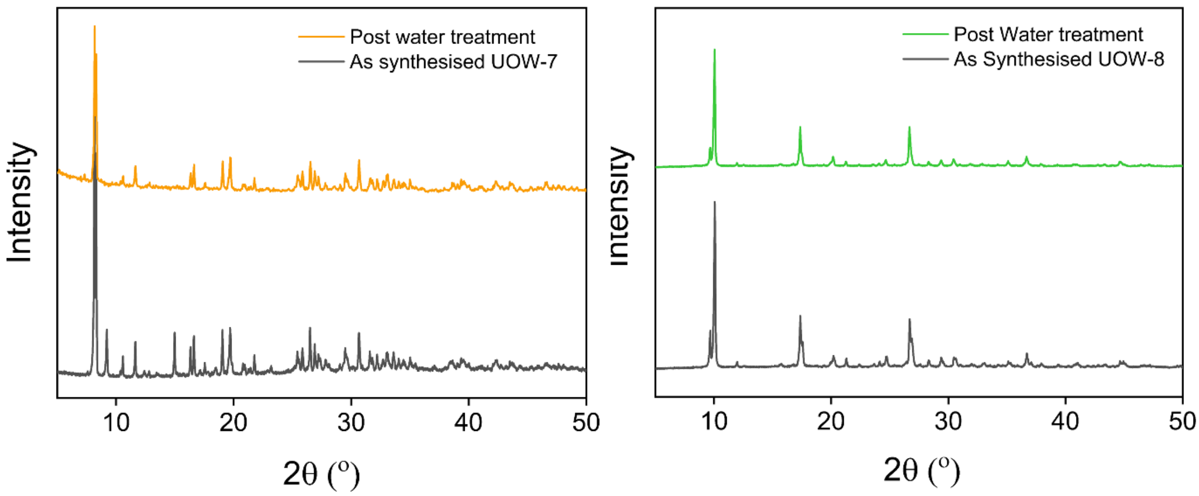

Figure S17. X-ray diffraction plot post water stability test of UOW-7 and UOW-8, respectively.

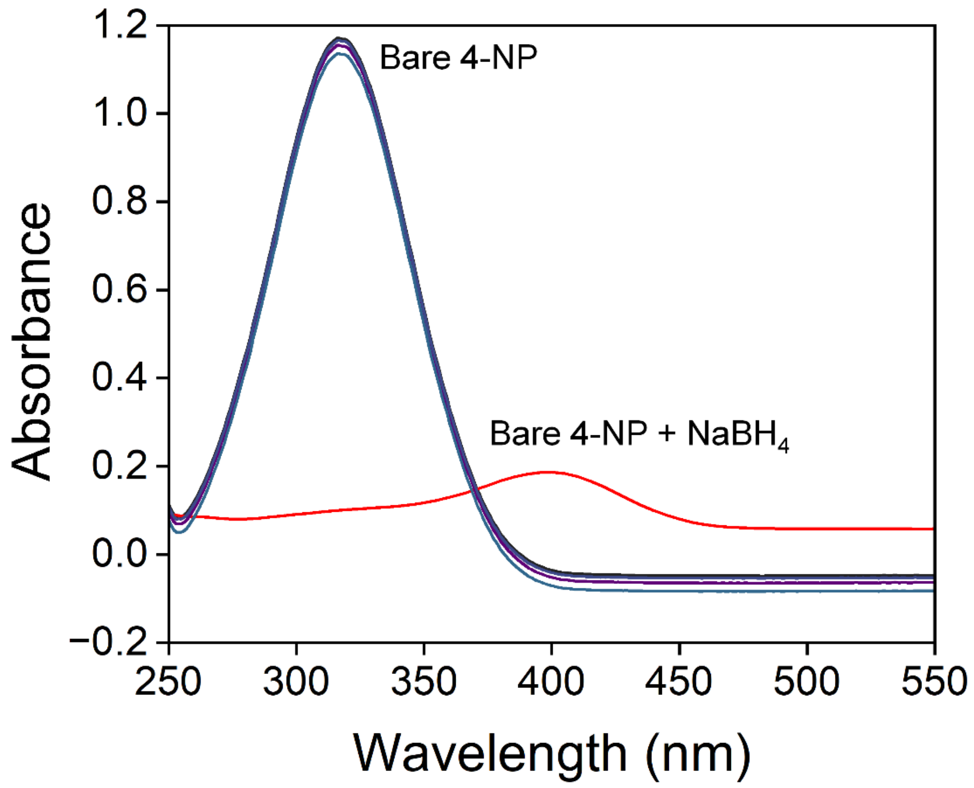

Figure S18. UV-Vis spectra of bare 4-Nitrophenol for several runs and 4- Nitrophenol with addition of $NaBH_4$

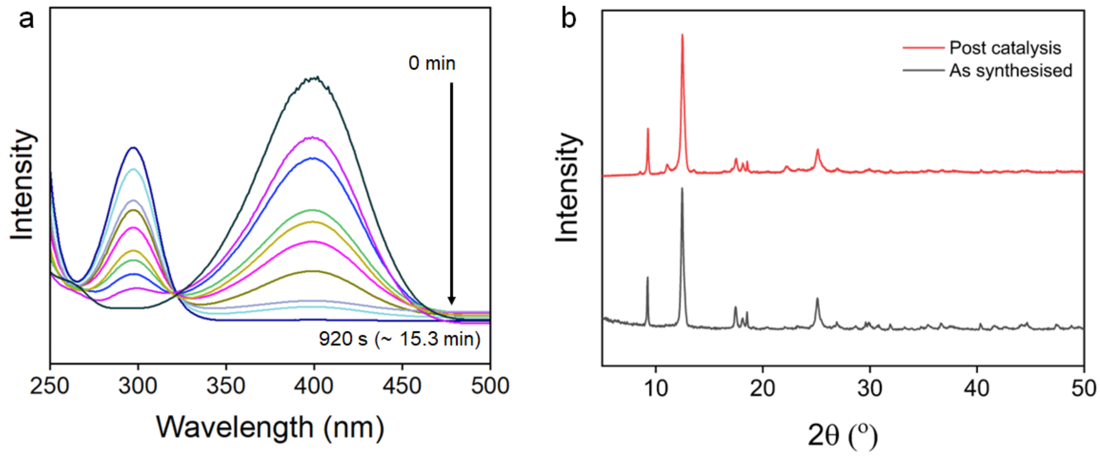

Figure S19. Time-dependent UV-vis spectral changes in p-nitrophenol and corresponding XRD of MIL-53 MOF before and post catalysis

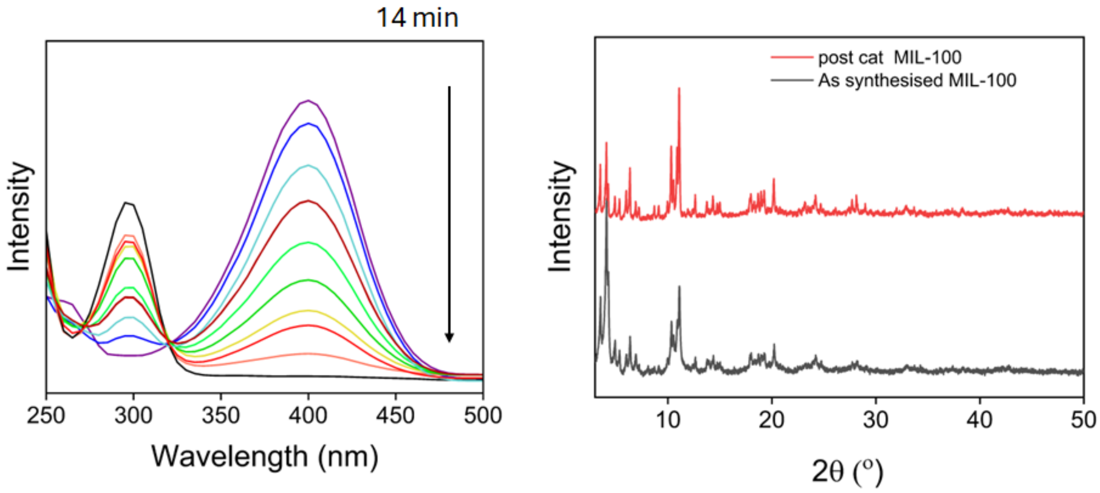

Figure S20. Time-dependent UV-vis spectral changes in *p*-nitrophenol and corresponding XRD of MIL-100 MOF before and post catalysis

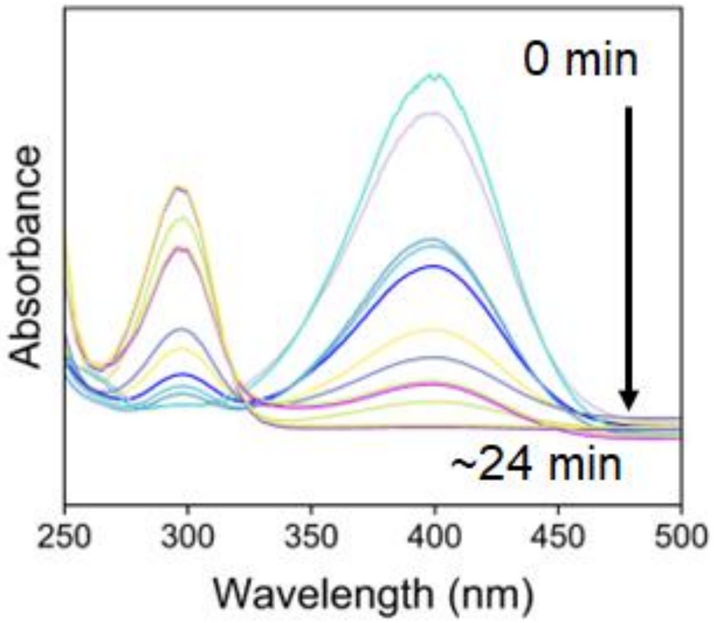

Figure S21. Time-dependent UV-vis spectral change for p-nitrophenol reduction using Fe_2O_3+ FDCA catalyst.

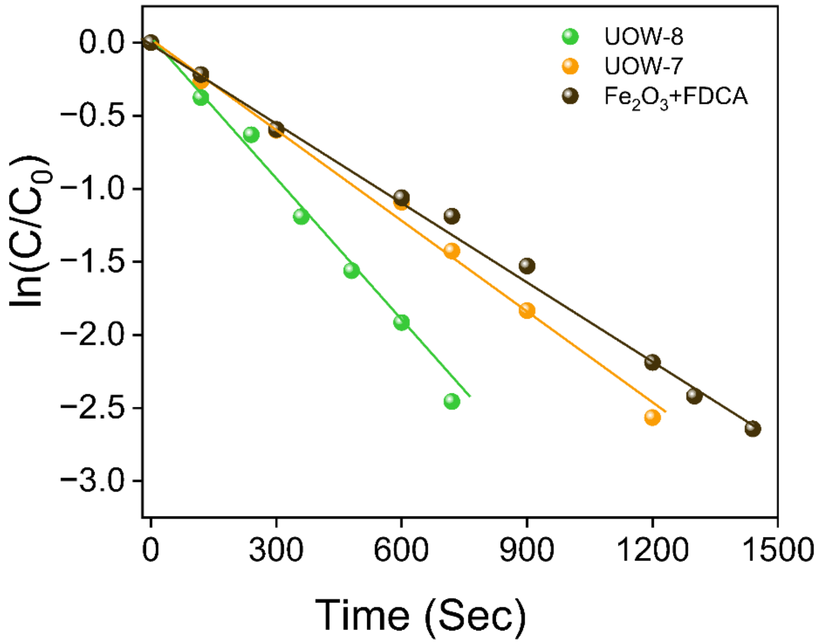

Figure S22. Semilogarithmic plots of 4-nitrophenol concentration *vs* time(*C* indicates reactant concentration, and C_0- indicates the initial concentration) for UOW-7 , UOW-8 and physical mix of Fe_2O_3 + FDCA at RT.

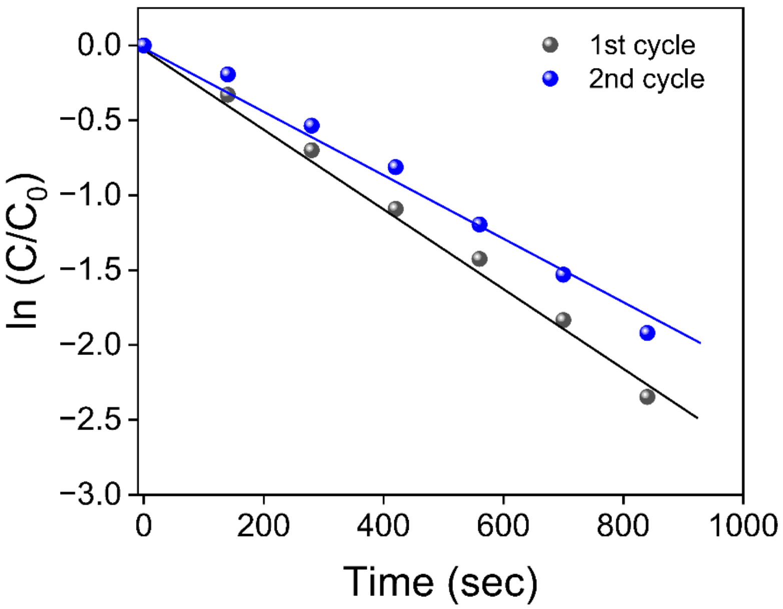

Figure S23: Semilogarithmic plots of 4-nitrophenol concentration *vs* time (*C* indicates reactant concentration, and C_0- indicates the initial concentration) for MIL-100 at RT after two cycles of catalysis.

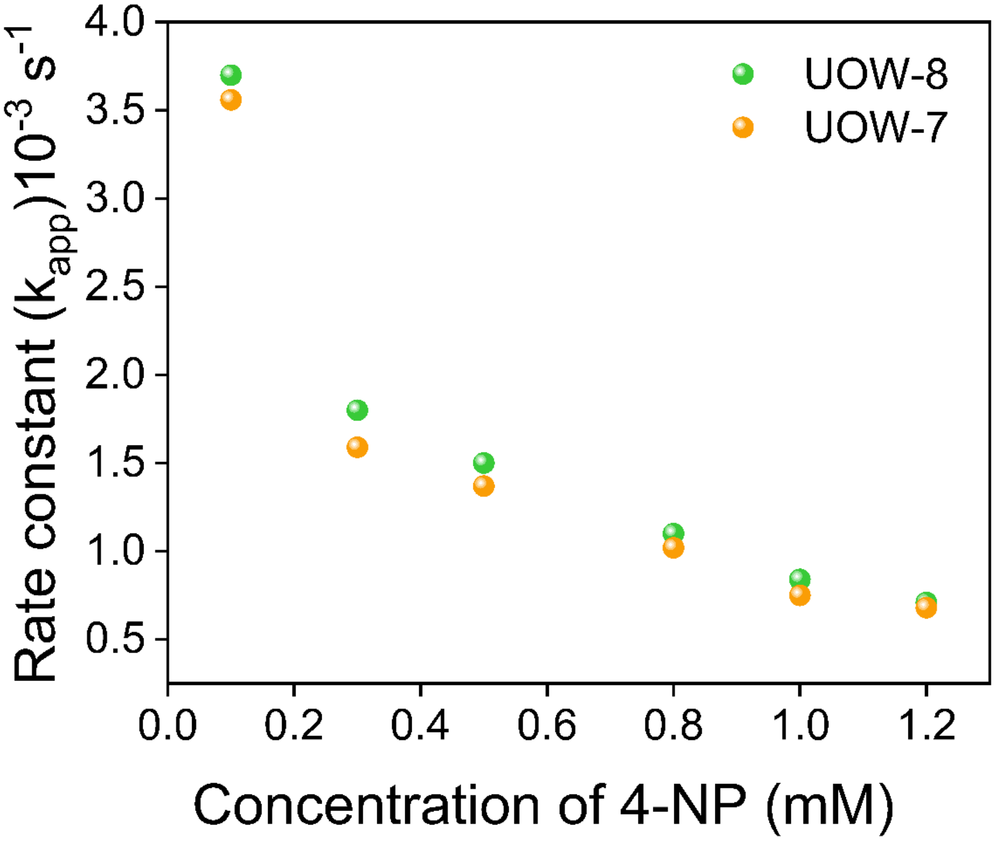

Figure S24. Plot of k_{app} *vs* 4-NP concentration in presence of the catalytic systems

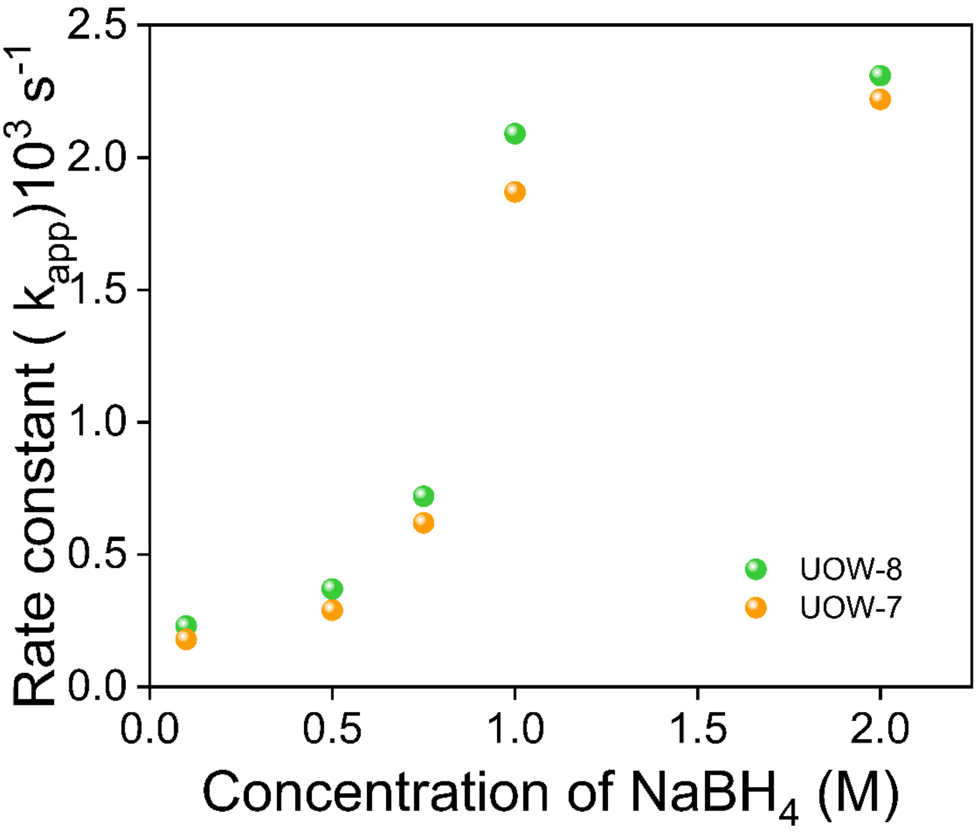

Figure S25. Plot of k_{app} *vs* $NaBH_4$ concentration in presence of the catalytic systems

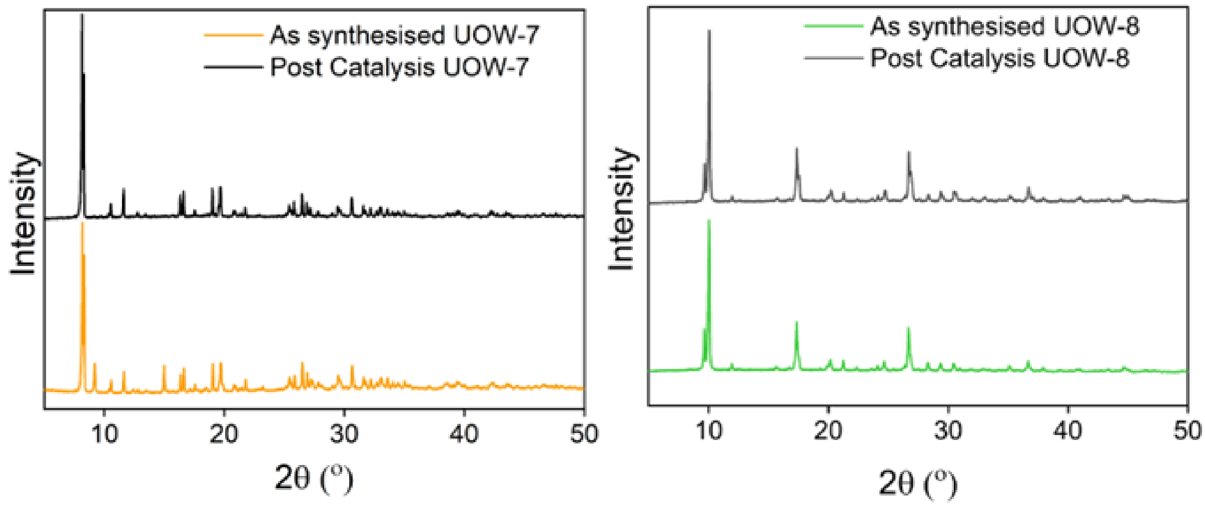

Figure S26. Post catalyst powder XRD of UOW-7 and UOW-8, respectively.

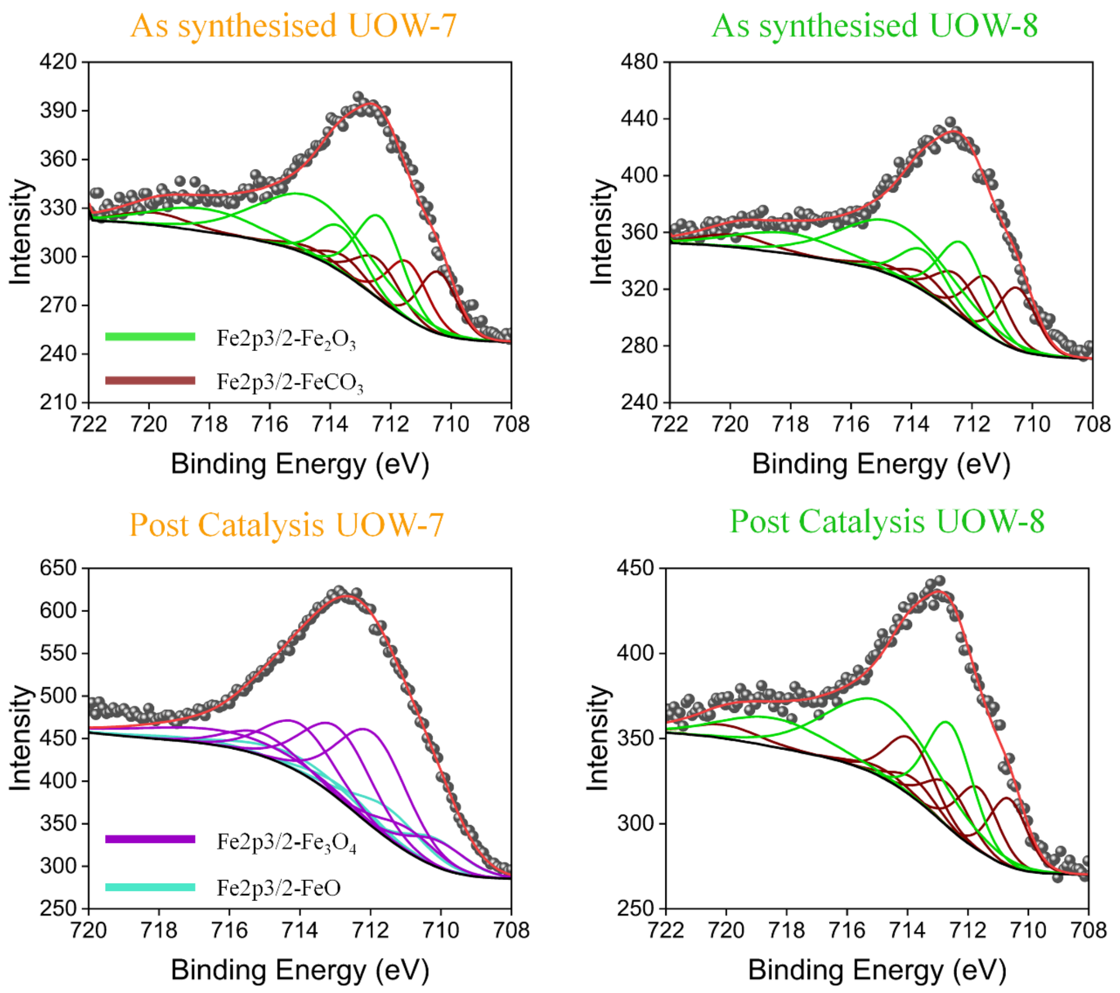

Figure S27. X-ray photoelectron spectra for as synthesised and post catalyst MOFs.

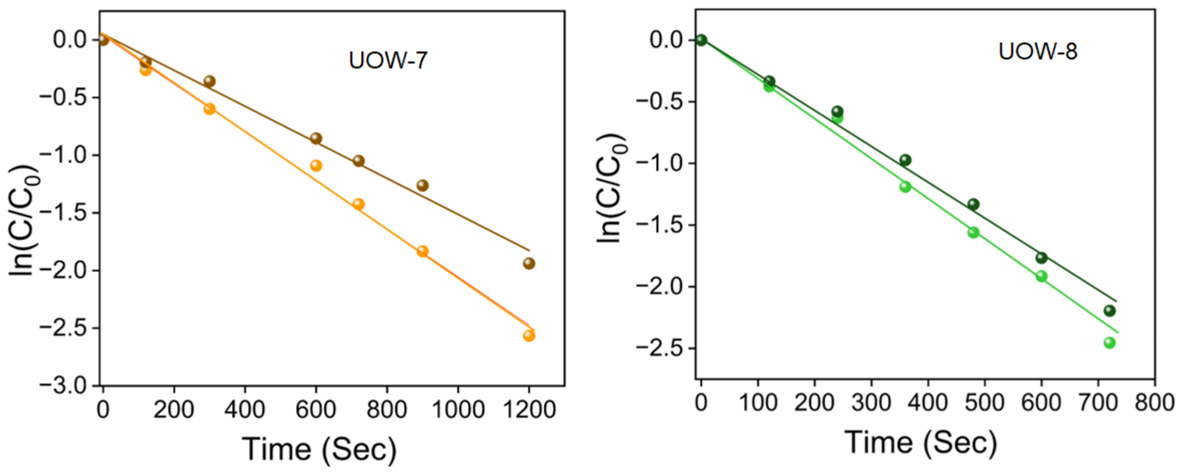

Figure S28. Comparison of kinetic parameter of UOW-and UOW-8 post 1st and 2nd cycle of catalysis at room temperature

Table S9. Data obtained from Eyring analysis, linear fits, and calculated rate coefficients for the nitrophenol reduction using UOW-7 catalyst at different temperatures.

Entry	*T* (K)	R^2	*k* (× 10^{-3} s^{-1})	ln (*k*/*T*)
1	273	0.97	0.735±0.076	-12.82
2	298	0.99	2.09±0.073	-11.91
3	323	0.98	2.77± 0.026	-11.66

Table S10. Data obtained from Eyring analysis, linear fits, and calculated rate coefficients for the nitrophenol reduction using UOW-8 catalyst at different temperatures.

0	*T* (K)	R^2	*k* (× 10^{-3} s^{-1})	ln (*k*/*T*)
1	273	0.98	1.63 ± 0.009	-12.03
2	298	0.99	3.39 ±0.012	-11.38
3	323	0.99	4.86 ±0.017	-11.10

Table S11. Calculated rate coefficients for the nitrophenol reduction using UOW-7, UOW-8 catalyst and Fe_2O_3+ FDCA at RT

Entry	*T* (K)	R^2	*k* (× 10^{-3} s^{-1})
1	298	0.98	2.09± 0.073
2	298	0.99	3.39 ±0.012
3	298	0.99	1.83 ± 0.045

Table S12. Transition state thermodynamic parameters: $\Delta H^{\neq}$ and $\Delta S^{\neq}$, determined using Eyring plots.

Entry	Catalyst	y = mx + c	R^2	$\Delta H^{\neq}$ (kJ mol^{-1})	$\Delta S^{\neq}$ (J mol^{-1} K^{-1})
1	UOW-7	2061.51x+5.17	0.96	17.14 (±5.14)	-240.52
2	UOW-8	1648.64x+5.94	0.98	13.71 (±2.77)	-246.93

Table S13. Gibbs free energy of the transition state determined for the catalysts.

Entry	System	Temperature (K)	$\Delta G^{\neq}$ (kJ mol^{-1})
1	UOW-7	273	82.80
		298	88.81
		323	94.83
2	UOW-8	273	81.11
		298	87.29
		323	93.46

Table S14. Comparison Chart of catalytic efficiency of UOW-7 and UOW-8 with respect to previously reported iron catalysts.

Catalyst	Time to achieve 50% conversion	Precious Metal used	Reference
PdMicrostructure-Fe_2O_3	3.25	Yes	8
Fe−N/Graphene nano shells	15	No	9
Fe_3O_4@ chitosan_AgNi	5	Yes	10
Bi^{3+}-doped Ni−Cu−Cr quinary ferrite nano-catalyst	10.5	No	11
Co_3O_4/$CoFe_2O_4$/α- Fe_2O_3	13.5	No	12
Ag/Fe_3O4	20	Yes	13
Au/Fe_2O_3	5	Yes	14
Au/Fe_3O_4	5	Yes	15
UOW-7	10	No	This Work
UOW-8	6	No	This Work

Table S15. XPS fitting parameters table for UOW-7.

UOW-7 as synthesised			UOW-7 post Catalysis		
Binding energy (eV)	% of region	Bonding environment	Binding energy (eV)	% of region	Bonding environment
710.43	9.8	Fe 2p3/2 - Fe_2O_3	710.18	5.8	Fe 2p3/2 - FeO
711.43	9.8	Fe 2p3/2 - Fe_2O_3	711.48	7.2	Fe 2p3/2 - FeO
712.43	7.3	Fe 2p3/2 - Fe_2O_3	712.68	3.5	Fe 2p3/2 - FeO
713.53	3.3	Fe 2p3/2 - Fe_2O_3	713.88	6.1	Fe 2p3/2 - FeO
714.63	1.8	Fe 2p3/2 - Fe_2O_3	717.18	1.4	Fe 2p3/2 - FeO
719.83	3.6	Fe 2p3/2 - Fe_2O_3	710.12	5.5	Fe 2p3/2 - Fe_3O_4
712.35	16.4	Fe 2p3/2 - $FeCO_3$	710.92	4.9	Fe 2p3/2 - Fe_3O_4
713.65	8.9	Fe 2p3/2 - $FeCO_3$	711.92	22.7	Fe 2p3/2 - Fe_3O_4
714.55	28.3	Fe 2p3/2 - $FeCO_3$	712.92	17	Fe 2p3/2 - Fe_3O_4
718.15	10.2	Fe 2p3/2 - $FeCO_3$	714.02	11.7	Fe 2p3/2 - Fe_3O_4
721.95	0.5	Fe 2p3/2 - $FeCO_3$	715.12	5.5	Fe 2p3/2 - Fe_3O_4
			716.22	8.7	Fe 2p3/2 - Fe_3O_4

Table S16. XPS fitting parameters table for UOW-8.

UOW-8 as synthesised			UOW-8 post catalysis		
Binding energy (eV)	% of region	Bonding environment	Binding energy (eV)	% of region	Bonding environment
710.49	10.5	Fe 2p3/2 - Fe_2O_3	710.66	9	Fe 2p3/2 - Fe_2O_3
711.49	10.5	Fe 2p3/2 - Fe_2O_3	711.66	9	Fe 2p3/2 - Fe_2O_3
712.49	7.8	Fe 2p3/2 - Fe_2O_3	712.66	6.6	Fe 2p3/2 - Fe_2O_3
713.59	3.5	Fe 2p3/2 - Fe_2O_3	713.76	3	Fe 2p3/2 - Fe_2O_3
714.69	2	Fe 2p3/2 - Fe_2O_3	714.86	1.7	Fe 2p3/2 - Fe_2O_3
719.89	3.9	Fe 2p3/2 - Fe_2O_3	720.06	3.3	Fe 2p3/2 - Fe_2O_3
712.3	15.8	Fe 2p3/2 - $FeCO_3$	712.6	17.2	Fe 2p3/2 - $FeCO_3$
713.6	8.6	Fe 2p3/2 - $FeCO_3$	713.9	9.4	Fe 2p3/2 - $FeCO_3$
714.5	27.2	Fe 2p3/2 - $FeCO_3$	714.8	29.7	Fe 2p3/2 - $FeCO_3$
718.1	9.8	Fe 2p3/2 - $FeCO_3$	718.4	10.7	Fe 2p3/2 - $FeCO_3$
721.9	0.5	Fe 2p3/2 - $FeCO_3$	722.2	0.5	Fe 2p3/2 - $FeCO_3$

Table S17. Kinetic rate constant post 1st and 2nd cycle of catalysis for UOW-7 and UOW-8 at RT, compared with MIL-100

Catalyst	Cycle	T (K)	R^2	k (× 10^{-3} s^{-1})
UOW-7	1st	298	0.99	2.09±0.073
	2nd	298	0.98	1.50 ±0.076
UOW-8	1st	298	0.99	3.39 ±0.012
	2nd	298	0.99	3.04±0.011
MIL-100	1st	298	0.99	2.76 ±0.08
	2nd	298	0.99	2.32±0.074

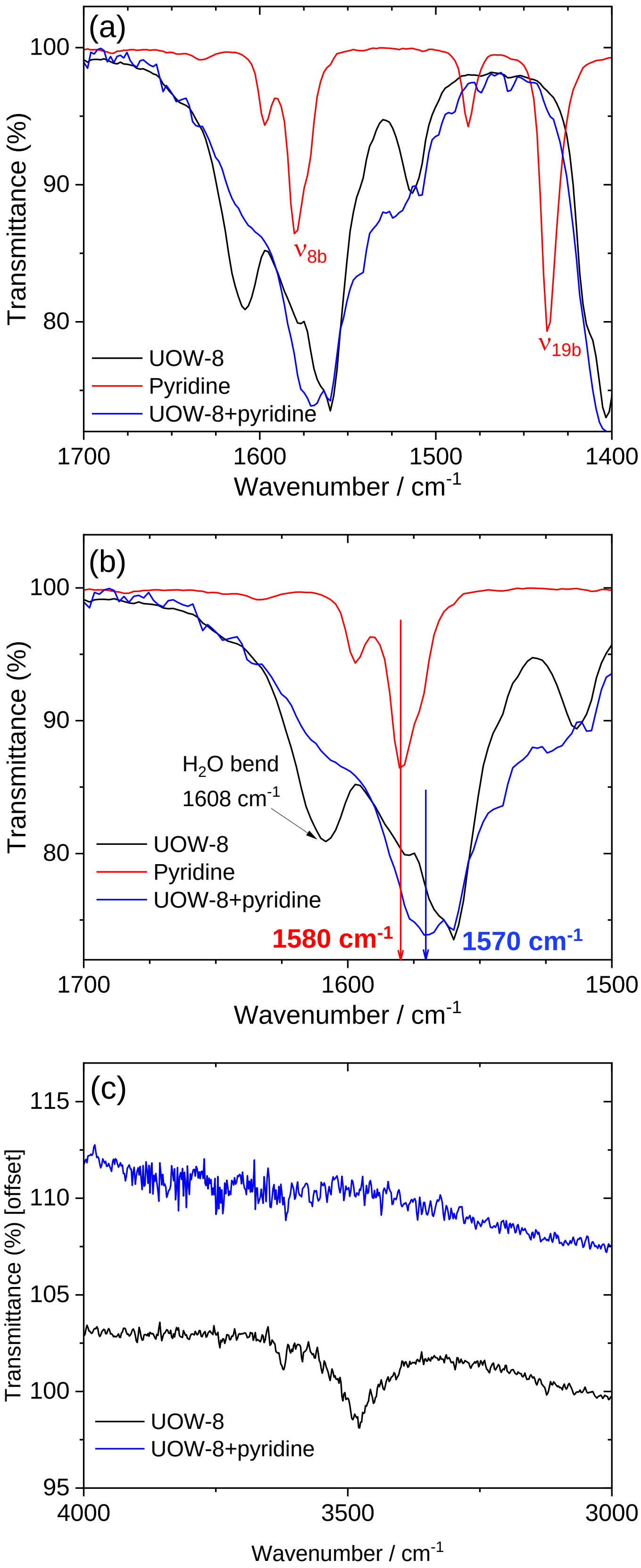

Figure S29: Infra-red spectra of pyridine adsorption on UOW-8 (a) characteristic bands of free pyridine showing its absence in the UOW-8 sample (b) assignment of characteristic bands in the adsorbed pyridine region and (c) the O-H stretch region confirming removal of water from the dried UOW-8 sample with pyridine adsorbed.

Table S18: Assignment of key bands in the IR spectra in Figure S29b

Sample	Band / cm^{-1}	Assignment	Reference(s)
Pyridine liquid	1580	ν_{8b}	[16]
UOW-8	1608	H-O-H bend	[17]
UOW-8 + pyridine	1570	Lewis-acid bound pyridine	[18,19]

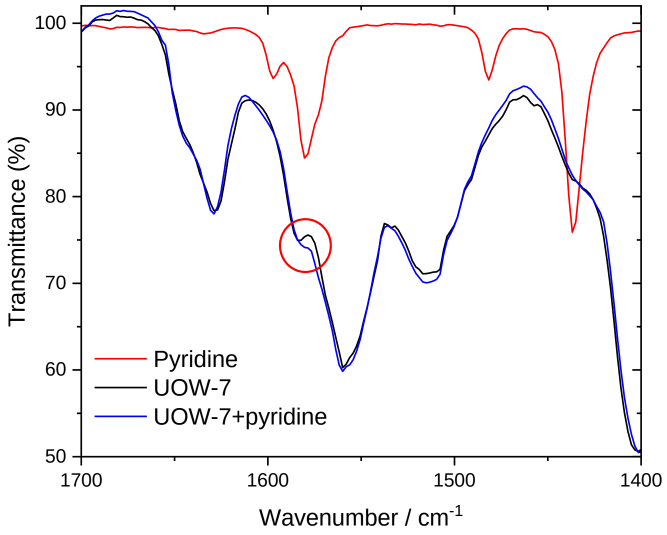

Figure S30: Infra-red spectra of pyridine adsorption on UOW-7 showing absence of any strong interaction with pyridine, with red circled region highlighting the region of interest. Note that the occluded water is not removed upon drying as seen by the H_2O bend at ~1620 cm^{-1}, confirming its entrapment in the structure.

References.

[1] E. P. Jahrman, W. M. Holden, A. S. Ditter, D. R. Mortensen, G. T. Seidler, T. T. Fister, S. A. Kozimor, L. F. J. Piper, J. Rana, N. C. Hyatt, M. C. Stennett, *Rev. Sci. Instrum.* 2019, **90**, 024106.

[2] B. Ravel, M. Newville, *Phys. Scr.* **2005**, 1007.

[3] G.M. Sheldrick, *Acta Cryst. A* **2015**, *71*, 3-8.

[4] L.J. Bourhis, O.V. Dolomanov, R.J. Gildea, J.A.K. Howard, H. Puschmann, *Acta Cryst. A* **2015**, *71*, 59-75.

[5] O.V. Dolomanov, L.J. Bourhis, R.J. Gildea, J.A.K. Howard, H. Puschmann, *J. Appl. Cryst.* **2009**,*42*, 339-341.

[6] A. Saha, S. S. Nia, J. A. Rodríguez, *Chem. Rev.* **2022**, *122*, 13883.

[7] N. E. Brese, M. O'Keeffe, *Acta Crystallogr. B.* **1991**, *47*, 192–197.

[8] S. Manivannan, S. An, J. Jeong, M. Viji, K. Kim, *ACS Appl. Mater. Interfaces* **2020**, *12*, 17557–17570

[9] T. Ma, X. Tan, Q. Zhao, Z. Wu, F. Cao, J. Liu, X. Wu, H. Liu, X. Wang, H. Ning, M. Wu, *Ind. Eng. Chem. Res.* **2020**, *59*, 129–136.

[10] R. Antony, R. Marimuthu, R. Murugavel, *ACS Omega* **2019**, *4*, 9241–9250.

[11] S. Singh, D. Singh, *ACS Sustainable Resour. Manage.* **2024**, *1*, 328–343.

[12] J.-L. Ortiz-Quiñonez, U. Pal, *ACS Omega* **2019**, *4*, 10129–10139.

[13] J.-R. Chiou, B.-H. Lai, K.-C. Hsu, D.-H. Chen, *J Hazard. Mater.* **2013**, *248–249*, 394–400.

[14] G. K. Larsen, W. Farr, S. E. Hunyadi Murph, *J. Phys. Chem. C* **2016**, *120*, 15162–15172.

[15] F. Lin, R. Doong, *J. Phys. Chem. C* **2011**, *115*, 6591–6598.

[16] K.N. Wong, S. D. Colson, *J. Mol. Spectrosc.*, **1984**, *104*, 129-151.

[17] M. Falk, *Spectrochim. Acta A*, **1982**, 40, 43-48.

[18] T. Barzetti, E. Selli, D. Moscotti L. Forni, *J. Chem. Soc., Faraday Trans.*, 1996, **92**, 1401-1407.

[19] D. L. Burnett , R. Oozeerally , R. Pertiwi , T. W. Chamberlain , N. Cherkasov , G. J. Clarkson , Y. K. Krisnandi , V. Degirmenci and R. I. Walton , *Chem. Commun.*, 2019, **55** , 11446-11449.
